# Prediction of in-hospital mortality risk for patients with acute ST-elevation myocardial infarction after primary PCI based on predictors selected by GRACE score and two feature selection methods

**DOI:** 10.3389/fcvm.2024.1419551

**Published:** 2024-10-22

**Authors:** Nan Tang, Shuang Liu, Kangming Li, Qiang Zhou, Yanan Dai, Huamei Sun, Qingdui Zhang, Ji Hao, Chunmei Qi

**Affiliations:** Department of Cardiology, The Second Affiliated Hospital of Xuzhou Medical University, Xuzhou, Jiangsu, China

**Keywords:** in-hospital mortality, percutaneous coronary intervention, ST-elevation myocardial infarction, global registry of acute coronary events, machine learning prediction, feature selection

## Abstract

**Introduction:**

Accurate in-hospital mortality prediction following percutaneous coronary intervention (PCI) is crucial for clinical decision-making. Machine Learning (ML) and Data Mining methods have shown promise in improving medical prognosis accuracy.

**Methods:**

We analyzed a dataset of 4,677 patients from the Regional Vascular Center of Primorsky Regional Clinical Hospital No. 1 in Vladivostok, collected between 2015 and 2021. We utilized Extreme Gradient Boosting, Histogram Gradient Boosting, Light Gradient Boosting, and Stochastic Gradient Boosting for mortality risk prediction after primary PCI in patients with acute ST-elevation myocardial infarction. Model selection was performed using Monte Carlo Cross-validation. Feature selection was enhanced through Recursive Feature Elimination (RFE) and Shapley Additive Explanations (SHAP). We further developed hybrid models using Augmented Grey Wolf Optimizer (AGWO), Bald Eagle Search Optimization (BES), Golden Jackal Optimizer (GJO), and Puma Optimizer (PO), integrating features selected by these methods with the traditional GRACE score.

**Results:**

The hybrid models demonstrated superior prediction accuracy. In scenario (1), utilizing GRACE scale features, the Light Gradient Boosting Machine (LGBM) and Extreme Gradient Boosting (XGB) models optimized with BES achieved Recall values of 0.944 and 0.954, respectively. In scenarios (2) and (3), employing SHAP and RFE-selected features, the LGB models attained Recall values of 0.963 and 0.977, while the XGB models achieved 0.978 and 0.99.

**Discussion:**

The study indicates that ML models, particularly the XGB optimized with BES, can outperform the conventional GRACE score in predicting in-hospital mortality. The hybrid models' enhanced accuracy presents a significant step forward in risk assessment for patients post-PCI, offering a potential alternative to existing clinical tools. These findings underscore the potential of ML in optimizing patient care and outcomes in cardiovascular medicine.

## 1 Introduction

Cardiovascular disease (CVD) constitutes a dominant global health challenge, particularly accentuated within low- and middle-income countries (LMICs). The growing prevalence of CVD risk factors within these regions obviously increases the burden of mortality associated with this disease (1–3). Myocardial infarction (MI) is a severe medical condition stemming from a sudden reduction in blood flow to the heart, resulting in tissue damage. Clinical manifestations typically include chest pain, shortness of breath, and weakness (4, 5). Preventative measures mostly contain lifestyle changes and pharmacological interventions (6). Treatment modalities include the management of beta-blockers, diuretics, ACE inhibitors, calcium channel blockers, and nitrates.

The effective management of ST-segment elevation myocardial infarction (STEMI) is considered important in inpatient care, a fact emphasized by the guidance provided in the 2012 and 2017 ESC Guidelines. These guidelines prioritize early reperfusion therapy, particularly through main percutaneous coronary intervention (PCI), for optimal STEMI treatment. The diagnosis of STEMI poses challenges due to its potential to represent conditions, requiring careful consideration of various clinical factors during electrocardiogram interpretation (7, 8). Furthermore, STEMI rises as a complication of infective endocarditis, associated with a distinguished 30-day mortality rate (9). Timely diagnosis and immediate restoration of blood flow, preferably through primary PCI, are key steps in reducing myocardial damage and preventing complications following STEMI (10).

Despite the developments in PCI technologies, the in-hospital mortality (IHM) subsequent to PCI in emergency cases persists at a remarkably high rate. A study conducted by Moroni (11) clarified that IHM often correlates with pre-existing serious cardiovascular conditions, with procedural complications attributing to a minority of cases. This suggests an imperative for enhanced treatment modalities for severe cardiovascular situations, particularly in addressing cardiogenic shock. However, the utility of procalcitonin (PCT) as a prognostic indicator in these conditions remains controversial. Covino et al. (12) observed that early assessment of PCT in patients with intra-abdominal infection (IAI) did not yield a significant impact on IHM, while Dutta et al. (13) highlighted the potential of PCT levels in predicting mortality in disapprovingly ill surgical patients. Within the spectrum of STEMI, Dawson et al. (14) reported a lack of substantial reduction in IHM despite changes in technical characteristics. These findings emphasize the demand for further research activities and targeted interventions aimed at justifying IHM following PCI in emergency scenarios.

In contemporary clinical practice, a multitude of risk grading tools are employed to assess the risk of IHM among patients. Notable among these are the History, Electrocardiogram, Age, Risk factors, initial Troponin (HEART) score, the Thrombolysis in Myocardial Infarction (TIMI) score, and the Global Registry of Acute Coronary Events (GRACE) score, as identified by Liu (15). Nevertheless, the efficacy of these tools can fluctuate across diverse patient populations, with certain instruments demonstrating suboptimal performance in present-day practice (16). Within main care backgrounds, there is an observable trend toward utilizing routine healthcare data for risk grading. However, comprehensive documentation regarding the specific tools applied and their performance remains lacking (17). Additionally, within the intensive care situation, there is incredulity regarding the relevance and reliability of scales employed to measure job stressors. This underscores the imperative for further investigation and scholarly inquiry in this domain (18).

Regarding the GRACE scale, despite advancements in treatment approaches, it continues to be a critical tool for evaluating the risk of adverse outcomes in cases of serious coronary syndromes (19). Continuous monitoring of mortality rates in coronary care elements using the GRACE score indicates that while it generally performs adequately, there are still areas where improvements can be made (20). Additionally, research has shown that the GRACE score is effective in predicting major cardiac events in patients presenting with chest pain and suspected acute coronary syndrome (21). Moreover, a modified version of the GRACE score, identified as the angiographic GRACE score, has been developed and validated as a beneficial tool for predicting IHM, specifically in Japanese patients with acute myocardial infarction (22).

Over the past few decades, Data Mining (DM) and Machine Learning (ML) have emerged as influential tools in medicine, particularly in predicting and diagnosing cognitive diseases (23). These methods have been applied to a wide variety of medical conditions, including type 2 diabetes, hypertension, cardiovascular disease, renal diseases, liver diseases, mental illness, and child health (24). The usage of ML in medical informatics has seen a significant increase, with Support Vector Machine (SVM) and Random Forest (RF) being the most popular algorithms for classification problems (25). However, there is no single algorithm that is universally suitable for diagnosing or predicting diseases, and the combination of different processes often yields the greatest results (26).

ML models have shown potential in predicting IHM following PCI in patients with serious STEMI. Studies conducted by Li (27) and Yang (28) employed data from the Chinese Acute Myocardial Infarction (CAMI) registry to develop prediction models, achieving both high performance and interpretability. Moreover, Deng (29) applied a RF algorithm to forecast both no-reflow and IHM in STEMI patients undergoing key PCI, demonstrating superior discrimination. Falcao (30) identified predictors of IHM in patients with STEMI undergoing pharmacoinvasive treatment, including age, comorbidities, and practical success. Additionally, Tanık (31) found that the PRECISE-DAPT score, a predictive tool for bleeding risk, was independently associated with IHM in STEMI patients undergoing primary PCI. Furthermore, Bai (32) compared the performance of various ML models in predicting 1-year mortality in STEMI patients with hyperuricemia, with the CatBoost model showing the highest accuracy. To validate the accuracy of ML models, particularly the XGBoost model, in predicting 1-year mortality in patients with anterior STEMI, Li (33) conducted further research. Collectively, these studies highlight the significant potential of ML in improving risk prediction for STEMI patients post-PCI, offering valuable insights into prognosis and treatment strategies. However, there is a gap in existing literature related to ML prediction model development based on imperative features of patients rather than those four parameters leading to GRACE score development. Also, integrating currently developed optimization algorithms for enhanced prediction accuracy by hybrid and ensemble approaches are the innovative methods which their absence is strongly felt in the literature review.

This study aims to introduce a new approach to investigate the risk factors contributing to IHM in patients with MI following PCI, applying advanced ML techniques. The research methodology involved gathering datasets related to various features of patients to assess their impact on the mortality risk of patients utilizing classifiers like Extreme Gradient Boosting (XGB), Light Gradient Boosting (LGB), Stochastic Gradient Boosting (SGB), and Histogram Gradient Boosting (HGB). Monte Carlo Cross-Validation (MCCV) was used to select the best prediction models based on their Accuracy. Techniques, for instance, Recursive Feature Elimination (RFE) and Shapley Additive Explanations (SHAP), were employed to identify important features for classification. Three different scenarios were designed to predict the risk of IHM within 30 days to provide clinicians with an estimate of patient survivability or mortality likelihood pre-treatment. The first scenario studies the efficacy of the traditional GRACE scale system (including Age, patient age, heart rate (HR), systolic blood pressure (SBP), and acute heart failure (AHF) class), widely entrenched within hospital protocols. The second and third scenarios employ a subclass of features selected via the Shapley Additive explanations (SHAP) and Recursive Feature Elimination (RFE) methods, respectively. All analysis conducted in Python programming software. By comparing the prediction performance of base single models and their hybrid framework (optimized with meta-heuristic algorithms such as Augmented Gray Wolf Optimizer (AGWO), Bald Eagle Search Optimization (BES), Golden Jackal Optimizer (GJO), and Puma Optimizer (PO)) utilizing these scenarios, the study aims to give valuable insights to enhance risk assessment strategies and patient care paradigms for MI patients undergoing PCI intervention.

## 2 Classification and model selection based on machine learning techniques

**Figure 1:**
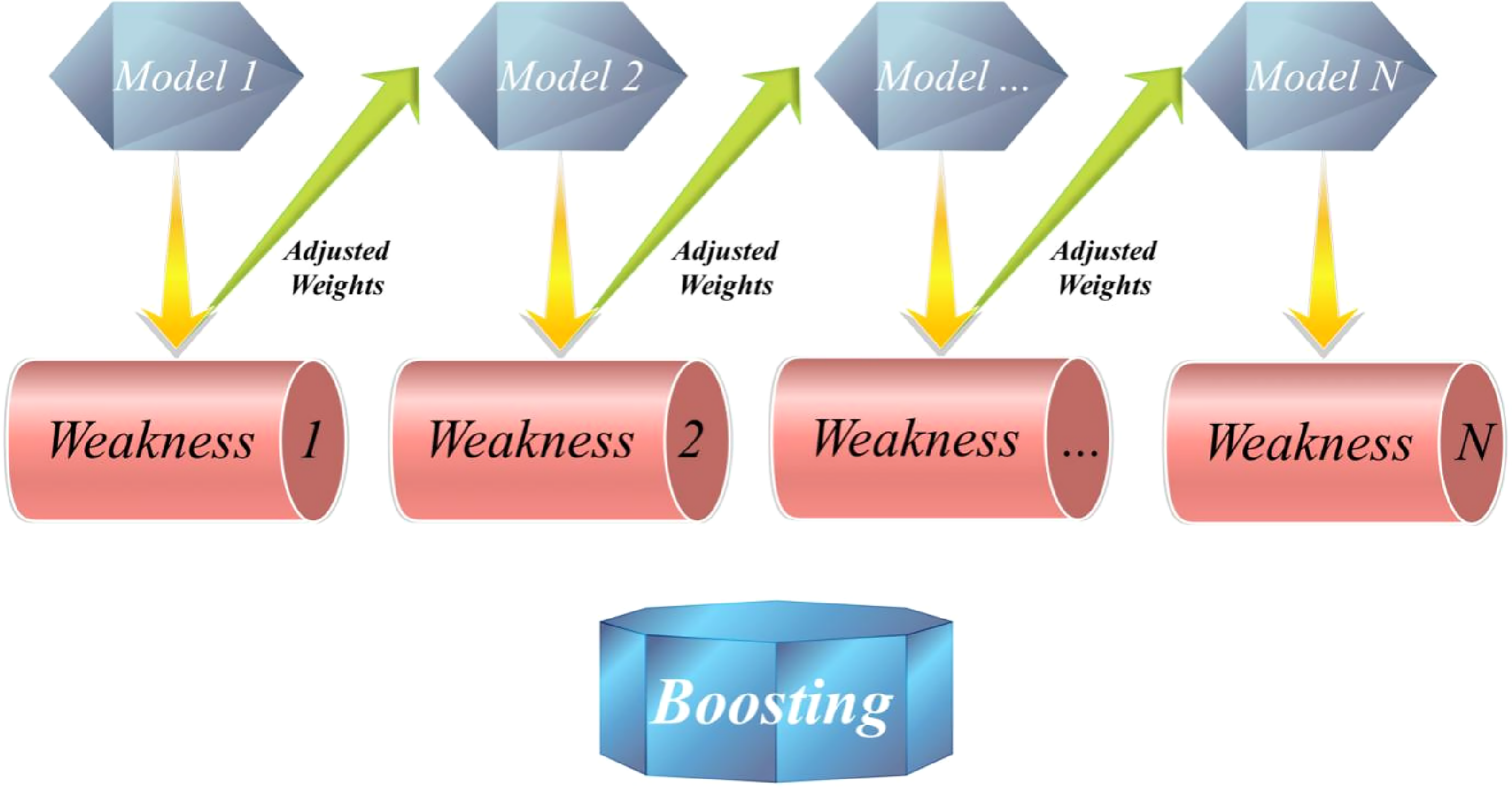
Boosting approach in ML.

The boosting approach involves utilizing a “weak” or “base” learning algorithm repeatedly, each time with a different subset of training examples (or a varied distribution or weighting over the examples). In each iteration, the base learning algorithm generates a new weak prediction rule. After numerous rounds, the boosting algorithm combines these weak rules into a single prediction rule, aiming for a substantially improved level of accuracy compared to any individual weak rule (Figure 1). This iterative process enhances the overall predictive power of the model (34).

### 2.1 Extreme Gradient Boost (XGB)

The Extreme Gradient Boost Classifier (XGBC) represents a sophisticated implementation of the gradient boosting technique, employing an ensemble approach to combine multiple sets of base learners (trees) to establish a strong model capable of making significant predictions (35). XGBC offers various advantages, including the ability to leverage parallel processing for improved computational efficiency, providing flexibility in setting objectives, incorporating built-in cross-validation, and effectively addressing splits in the presence of negative loss. With these advantages, XGBC emerges as a highly suitable choice for analyzing classification data. Applying a tree-based methodology, XGBC constructs decision trees to classify training data, facilitating the achievement of specific target outcomes (36).

The gradient boosting procedure encompasses the subsequent sequential steps:

• The initialization of the boosting algorithm involves the description of the function $F_{0}(x)$ (Equation 1):
  (1) $$F_{0}(x)=\arg\;\mathrm{mi}n_{ℸ}\sum_{i-1}^{n}L(y_{i},ℸ)$$
• The iterative calculation includes the derivation of the gradient of the loss function (Equation 2):
  (2) $$r_{im}=-\alpha {\left[\frac{\partial (L(y_{i},F(X_{i})))}{\partial F(x_{i})}\right]}_{F(X)=F_{m-1}(x)}$$

Where *α* is the learning rate.

• Subsequently, each hm(x) is fitted based on the gradient developed at each iterative step:
• The purpose of the multiplicative factor $y_{m}$ for each terminal node is executed, and subsequently, the boosted model Fm(x) is formulated (Equation 3):
  (3) $$F_{m}(x)=F_{m-1}(x)+{ℸ}_{m}h_{m}(x)$$

### 2.2 Light Gradient Boosting (LGB)

LGB is a rapid training outline that blends decision tree algorithms with boosting methods. It prioritizes speed, using histogram-based techniques to accelerate training and conserve memory (37). Different from traditional trees, LGB employs leaf-wise tree growth, efficiently identifying high-branching gain leaves to optimize performance (38, 39).

The calculation procedures of LGB, delineated step by step in (40), involve finding a projected function $\hat{\;f}\;(x)$ that approximates the function $f^{*}(x)$ based on the given training dataset $X=\;\{\left(\;x_{i},\;y_{i}\;\right){\}}_{i=1}^{m}$. The primary objective is to minimize the expected values of specific loss functions, signified as L(y,f(x)) (Equation 4).

(4) $$\hat{\;f}(x)\arg\underset{f}{\min}E_{y,x}\;L(y,f(x))$$

In the process of approximating the final model, LGB will integrate a combination of multiple regression trees, represented as $\overset{T}{\underset{t=1}{\sum }}f_{t}(x)$ (Equation 5).

(5) $$f_{T}(X)=\overset{T}{\underset{t=1}{\sum }}f_{t}(X)$$

The regression trees signified as $w_{q}\;(x),\;q\;\in \{1,\;2,\ldots ,N\;\}$, denote decision rules, where *N* is the number of leaves in each tree. *q* signifies the decision rule, and *w* is a vector representing the weights of the leaf nodes. The model is incrementally trained at step t in an additive manner.

(6) $$\Gamma_{t}≅\overset{N}{\underset{\;j=1}{\sum }}L(y_{i},\;F_{t-1}(x_{i})+f_{t}(x_{i}))\;$$

The Newton's method is employed to rapidly estimate the objective function, and (Equation 6) is simplified by eliminating the constant term:

(7) $$\Gamma_{t}≅\overset{N}{\underset{\;j=1}{\sum }}(g_{i}f_{t}(x_{i})+\frac{1}{2}h_{i}f_{t}^{2}(x_{i}))\;$$

In the given equation, $g_{i}$ and $h_{i}$ denote the first- and second-order gradient statistics of the loss functions. If the sample set for leaf *j* is denoted as $I_{j}$, then (Equation 7) can be transformed into (Equation 8):

(8) $$\Gamma_{t}=\overset{J}{\underset{\;j=1}{\sum }}((\underset{i\epsilon I_{j}}{\sum }g_{i})\omega_{j}+\frac{1}{2}(\underset{i\epsilon I_{j}}{\sum }h_{i}+\lambda )\omega_{j}^{2})\;$$

Equations 9, 10 are employed to calculate the optimal leaf weights $\omega_{j}^{*}\;$ and the extreme values of $\Gamma_{K}$ concerning the tree structure q(x):

(9) $$\omega_{j}^{*}=-\frac{\sum_{i\epsilon I_{j}}g_{i}}{\sum_{i\epsilon I_{j}}h_{i}+\lambda }\;$$

(10) $$\Gamma_{T}^{*}=-\frac{1}{2}\overset{J}{\underset{\;j=1}{\sum }}\frac{{\left(\sum_{i\epsilon I_{j}}g_{i}\right)}^{2}}{\sum_{i\epsilon I_{j}}h_{i}+\lambda }\;$$

The term $\omega_{j}^{*}$ signifies the weight function assessing the effectiveness of the tree structure q(x). Ultimately, the objective function is derived by consolidating the splits.

(11) $$G=\;\frac{1}{2}\left(\frac{{\left(\sum_{i\epsilon I_{l}}g_{i}\right)}^{2}}{\sum_{i\epsilon I_{l}}h_{i}+\lambda }+\frac{{\left(\sum_{i\epsilon I_{r}}g_{i}\right)}^{2}}{\sum_{i\epsilon I_{r}}h_{i}+\lambda }+\frac{{\left(\sum_{i\epsilon I}g_{i}\right)}^{2}}{\sum_{i\epsilon I}h_{i}+\lambda }\right)$$

The objective function is defined as the sum of the splits with $I_{l}\;$ and $I_{r}$ representing the samples in the left and right branches, respectively (Equation 11).

### 2.3 Histogram-based Gradient Boosting (HGB)

Histograms are valuable tools for visualizing data distribution and frequency, especially with repetitive data. Grouping input data into bins, as in histograms, enhances model flexibility. Combining histogram-based methods with gradient boosting leads to strong ML ensembles, yielding high-performance models (41). HGBoost employs numeral-based data structures like histograms instead of sorted continuous values during tree-building, enabling it to capture complex nonlinear relationships in datasets effectively. This integration of gradient boosting with histogram-based techniques allows HGBoost to excel in modeling and optimizing feature connections (42).

Histogram-based Gradient Boosting Classification (HGBC) represents a difficult iteration of gradient boosting, employing decision trees as fundamental models and leveraging histograms to achieve outstanding improvements in computational efficiency. Observed remarks show that this methodology yields superior outcomes, diminishes ensemble size, and expedites inference, rendering it an attractive proposition for tackling intricate datasets within academic investigations (43).

### 2.4 Stochastic Gradient Boosting Machines (SGB)

Friedman (44) proposed Stochastic Gradient Boosting Machines (SGB), a method extensively employed in both classification and regression tasks. Decision stumps or regression trees serve as common choices for weak classifiers within SGB. The main aim of SGB is to train weak learners to minimize loss functions, such as mean square errors, with subsequent weak learners benefiting from the residuals of preceding ones for training.

Consequently, there is a reduction in the value of the loss function for the present weak learners. Employing the bagging technique serves to mitigate correlation among these learners, with each undergoing training on subsets sampled without replacement from the entirety of the dataset. The final prediction is then derived through the amalgamation of predictions generated by this cohort of weak learners (45).

### 2.5 Monte-Carlo cross-validation (MCCV) for model selection

Numerous methods, such as the Akaike information criterion (46) and $C_{p}$ statistics (47), tackle the task of model selection. Nevertheless, cross validation (CV) emerges as a standout approach (48–51), arranging a predictive perspective in this process. In CV, upon selecting a model (α), the *n* samples (referred to as S) undergo a division.

The initial component, identified as the calibration set ($S_{c}$), consists of $n_{c}$ samples applied for fitting the model, represented by the submatrix $X_{\alpha S_{c}}$ and sub-vector $Y_{S_{c}}$. The subsequent section termed the validation set ($S_{v}$), comprises $n_{v}=n-n_{c}$ samples dedicated to model validation, depicted by the submatrix $X_{\alpha S_{v}}$ and sub-vector $Y_{S_{v}}$. This arrangement leads to a total of $\left(\begin{array}{c} n\\ n_{v} \end{array}\right)$ possible sample divisions. In each division, the model is fitted using the $n_{c}$ samples from the standardization set $S_{c}$, resulting in the estimation ${\hat{\beta }}_{\alpha S_{c}}$. Treating the samples in the validation set as if they were future data points, the fitted model predicts the response vector $y_{S_{v}}$ (Equation 12).

(12) $${\hat{y}}_{\alpha S_{v}}=X_{\alpha S_{v}}^{t}{\hat{\beta }}_{\alpha S_{c}}$$

The Accuracy across all samples in the validation set is considered by (Equation 13):

(13) $$\mathrm{Accuracy}(S_{v},\alpha )=\frac{1}{n_{v}}||y_{S_{v}}-{\hat{y}}_{\alpha S_{v}}|{|}^{2}$$

The formula involves calculating the Euclidean norm of a vector within a framework where a set *S* is comprised of elements from various validation sets, each corresponding to different sample splits denoted as $\left(\begin{array}{c} n\\ n_{v} \end{array}\right)$. In this framework, the CV standard is defined by excluding a specific number of samples $n_{v}$ for validation, providing a method for systematically evaluating models on subsets of data.

(14) $$CV_{n_{v}}(\alpha )=\frac{\sum_{S_{v}\in S}\mathrm{Accuracy}(S_{v},\;\alpha )}{\left(\begin{array}{c} n\\ n_{v} \end{array}\right)}$$

For each α∈R, the computation of $CV_{n_{v}}(\alpha )$ is conducted. (Equation 14) serves as an estimate for Accuracy within the constraints of finite samples. The CV criterion is focused on identifying the optimal $\alpha^{*}$ that maximizes values across all $CV_{n_{v}}(\alpha )$ for α∈R. As a result, the model is characterized by variables indexed by the integers in $\alpha^{*}$ is chosen.

The widely used leave-one-out Cross-Validation (LOO-CV), where $n_{v}=1$, is extensively applied in chemometrics. However, research findings have shown that models selected through LOO-CV can be inaccurately asymptotic. Although LOO-CV can choose a model with a bias $b_{\alpha }=0\;$ that approaches infinity encompassing all non-zero elements in $\beta_{\alpha }$, it tends to include unnecessary additional variables in the model (52). This suggests that the model's dimension $P_{\alpha }$ is not optimally concise, potentially leading to overfitting concerns.

It has been established that, in general, CV, under the conditions $n_{c}\to \infty$ and $n_{v}/n\to 1$ (53), the likelihood of selecting the model with the best predictive capability tends toward unity when $n_{v}$ samples are reserved for validation. Consequently, the $CV_{n_{v}}(\alpha )$ benchmark (Equation 14) shows asymptotic consistency. Yet, practically computing $CV_{n_{v}}$ with a large $n_{v}$ is infeasible due to its exponential computational complexity. To tackle this issue, MCCV offers a simple and efficient solution. For a given *α*, the samples are randomly split into two sets: $S_{c}(i)$ (of size $n_{c}$) and $S_{v}(i)\;$ (of size $n_{v}$). This process is repeated *N* times, defining the repeated MCCV criterion as follows (Equation 15):

(15) $$\mathrm{MCC}V_{n_{v}}(\alpha )=\frac{1}{Nn_{v}}\overset{N}{\underset{i=1}{\sum }}\|y_{S_{v}(i)}-{\hat{y}}_{\alpha S_{v}(i)}{\|}^{2}$$

Employing the Monte Carlo method greatly decreases computational complexity. Theoretically, decreasing the number of samples for model calibration requires increasing the number of repetitions. Typically, it is deemed adequate to set $N=n^{2}$ to ensure that $\mathrm{MCC}V_{n_{v}}$ achieves similar performance to traditional $CV_{n_{v}}$ (54).

In this study, 70% of the samples were considered for the fitting (training) of the prediction models, 30% were allocated for the validation process (testing), and finally, two LGBM and XGBC models with an accuracy of 0.97 and 0.98 have been selected, and in the following, only these two models will be examined in their hybrid version.

## 3 Detailed data assessment

### 3.1 Data description and preprocessing

The study used data from patients treated at the Regional Vascular Center of Primorsky Regional Clinical Hospital in Vladivostok from 2015 to 2021. Patients were selected for inclusion in the STEMI and PCI study based on criteria confirmed upon their admission to the hospital. Exclusion criteria comprised non-ST elevation myocardial infarction, unconfirmed STEMI, or the absence of an indication for PCI. Finally, 4,677 patients were included in the study, from which 4,359 patients were in the “Alive” group who did not die within 30 days of the study after PCI, and 318 patients were in the “Die” group who died in hospital. The “Die” group comprised patients who passed away at any point during these 30 days, including those who did not survive to undergo post-PCI assessments. Conversely, the “Alive” group consisted of patients who survived the entire 30-day period and were monitored in the hospital throughout. It is important to note that patients with missing data were excluded from the dataset of those patients with no risk of death due to the abundance of information (the number of samples decreased to 2,709). For 318 patients who experienced IHM after PCI, the Multiple Imputation by Chained Equations (MICE) method was used to handle missing data. MICE achieves multiple imputation by creating multiple complete datasets, analyzing each dataset separately, and then combining the results to reduce the bias that a single imputation method might introduce (55). This method fully considers the uncertainty of the data when dealing with missing data, especially suitable for the complex multivariate data structure in this study. Compared with single imputation, MICE can provide more reliable statistical inference when dealing with a large amount of missing data. Ultimately, a cleaned dataset of 3,027 patients with 41 features, as described below in related categories, was chosen for the prediction task:

#### 3.1.1 Cardiovascular parameters

SPAP (Systolic Pulmonary Arterial Pressure), LVRMI (Left Ventricular Regional Motion Index), EF LV (Left Ventricular Ejection Fraction), ESV (End-Systolic Volume), LVRWTI (Left Ventricular Relative Wall Thickness Index), La1 (Left Atrial Diameter), Ra2 (Right Atrial Diameter), Ra1 (Right Atrium Pressure), PI (Pulsatility Index), EDV (End-Diastolic Volume), La2 (Left Atrial Pressure), SBP (Systolic Blood Pressure), DBP (Diastolic Blood Pressure).

#### 3.1.2 Blood parameters

NEUT (Neutrophils), EOS (Eosinophils), WBC (White Blood Cell count), Hb (Hemoglobin), RBC (Red Blood Cell count), PLT (Platelet count), LYM (Lymphocyte count).

#### 3.1.3 Coagulation parameters

TT (Thrombin Time), INR (International Normalized Ratio), APTT (Activated Partial Thromboplastin Time), PCT (Plateletcrit).

#### 3.1.4 Metabolic parameters

Urea [Blood Urea Nitrogen (BUN)], Glu (Glucose), Cr (Creatinine).

#### 3.1.5 Anthropometric parameters

Age (Patient's Age), Weight (Patient's Weight), Height (Patient's Height), BMI (Body Mass Index).

#### 3.1.6 Diagnostic parameters

Killip class [Killip Classification (classification of heart failure severity)], Form STEMI (STEMI Diagnosis), CKD (chronic kidney disease), AFib (Atrial Fibrillation), Diabetes (Diabetes Mellitus), COPD (Chronic Obstructive Pulmonary Disease), aMI (Acute Myocardial Infarction) And Sex (Patient's Gender).

### 3.2 Feature selection

#### 3.2.1 Shapley Additive Explanations (SHAP)

SHAP, a method for attributing features additively, draws from both game theory and local explanations (56). The Shapley value has gained popularity as a method for providing interpretable feature attribution in ML models (57). SHAP simplifies inputs by transforming the original inputs *x* into a simplified representation *z* through a mapping function $x=h_{x}(z)$. This simplification enables the approximation of the original model f(x) using a linear function of binary variables based on z (Equation 16):

(16) $$f(x)=g(z)=\varphi_{0}+\overset{M}{\underset{i=1}{\sum }}\varphi_{i}z_{i}$$

z is a binary vector with *M* elements representing input features, $\varphi_{0}$ denotes the attribution value of the model when *z* is all zeros, calculated as $f(h_{x}(0))$, and $\varphi_{i}$ represents the attribution value of the $i_{th}$ feature (Equations 17, 18).

(17) $$\varphi_{i}=\underset{S\in F/\{i\}}{\sum }\frac{|S|!(M-|S|!-1)!}{M!}[\;f_{x}(S\cup \{i\})-f_{x}(s)]$$

(18) $$f_{x}(S)=f(h_{x}^{-1}(Z))=E[\;f(x)|x_{s}]$$

SHAP stands out due to its three core properties: *local accuracy*, *consistency*, and *proficiency* in handling missing data. It uses the SHAP value $\varphi_{i}$ as a unified metric for additive feature attributions. In the SHAP framework, *F* represents the subset of non-zero inputs in *z*, while *S* indicates the subset of *F* obtained by excluding the $i_{th}$ feature (58). Known for its model-agnostic nature, SHAP shows impressive adaptability across various ML and DL models, effectively determining the relative importance of individual input features within additive feature attribution methodologies (59). Table 1 reports SHAP values obtained for each feature in the dataset based on each base models and selected features.

**Table 1:** SHAP values and selected features from the dataset based on each base model (scenario 2).

NO	Parameter	SHAP values (LGB)	Selected features (LGB)	SHAP values (XGB)	Selected features (XGB)
1	Sex	0.007		0.026	
2	Age	0.135		0.217	
3	Height	0.070		0.176	
4	Weight	0.073		0.205	
5	BMI	0.106		0.181	
6	SBP	0.041		0.091	
7	DBP	0.100		0.146	
8	PBP	0.052		0.090	
9	HR	0.291	***✓***	0.445	***✓***
10	Cr	0.088		0.189	
11	Killip class	0.147		0.259	***✓***
12	EF LV	0.216	***✓***	0.305	***✓***
13	EDV	0.068		0.102	
14	ESV	0.135		0.150	
15	LVRWTI	0.087		0.149	
16	LVRMI	0.328	***✓***	0.570	***✓***
17	SPAP	0.419	***✓***	0.493	***✓***
18	La1	0.082		0.159	
19	La2	0.064		0.111	
20	Ra1	0.080		0.130	
21	Ra2	0.081		0.147	
22	WBC	0.151		0.283	***✓***
23	NEUT	0.669	***✓***	0.886	***✓***
24	LYM	0.051		0.117	
25	EOS	0.345	***✓***	0.436	***✓***
26	RBC	0.117		0.259	
27	Hb	0.138		0.209	
28	PLT	0.085		0.118	
29	Glu	0.452	***✓***	0.574	***✓***
30	Urea	0.481	***✓***	0.651	***✓***
31	PCT	0.064		0.086	
32	PI	0.080		0.152	
33	INR	0.532	***✓***	0.684	***✓***
34	TT	0.554	***✓***	0.569	***✓***
35	APTT	0.067		0.179	
36	Form STEMI	0.033		0.052	
37	AFib	0.021		0.025	
38	Diabetes	0.007		0.025	
39	CKD	0.024		0.084	
40	aMI	0.004		0.000	
41	COPD	0.006		0.005	

**Figure 2:**
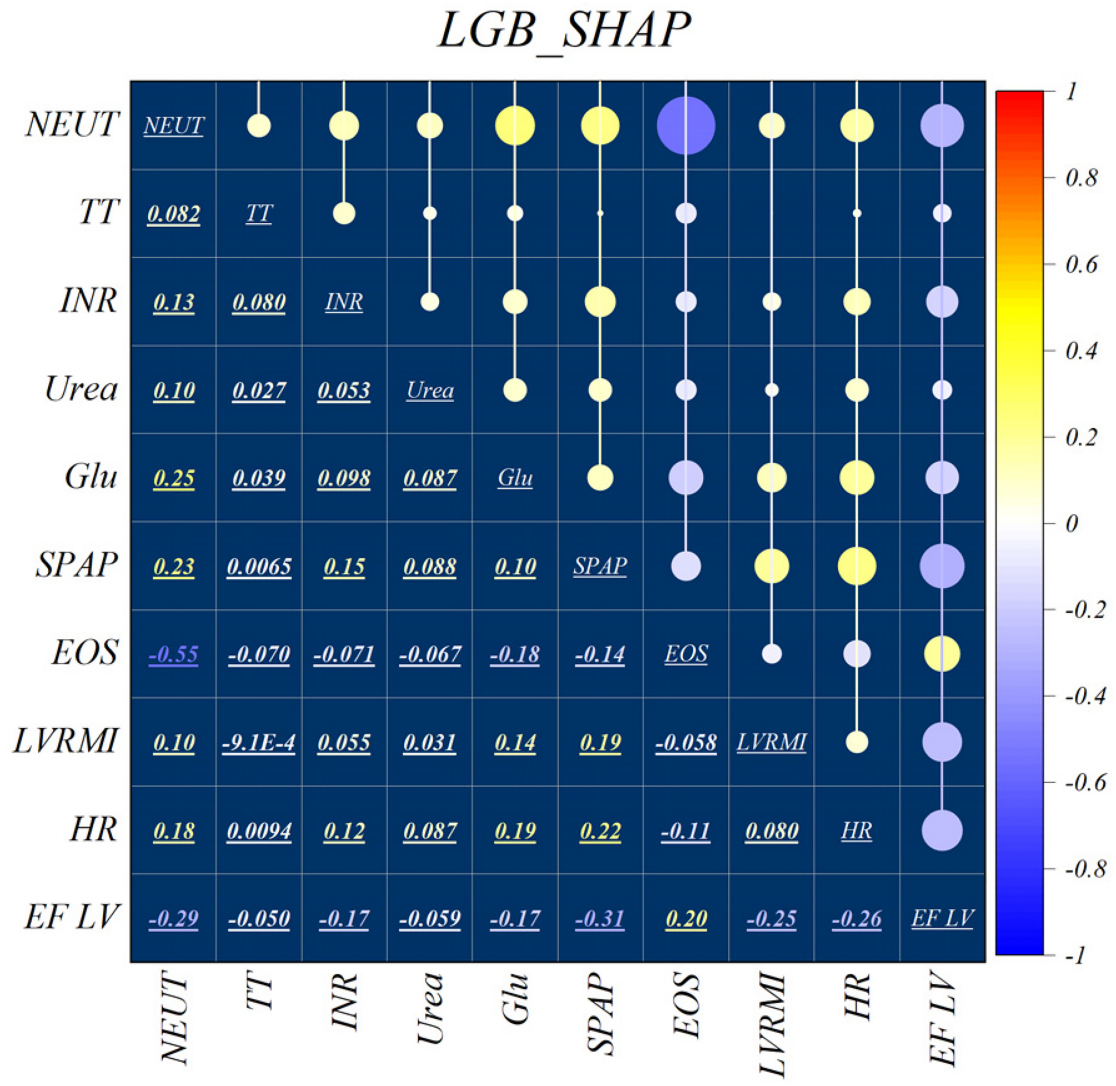
Feature selection and SHAP analysis for LGB.

**Figure 3:**
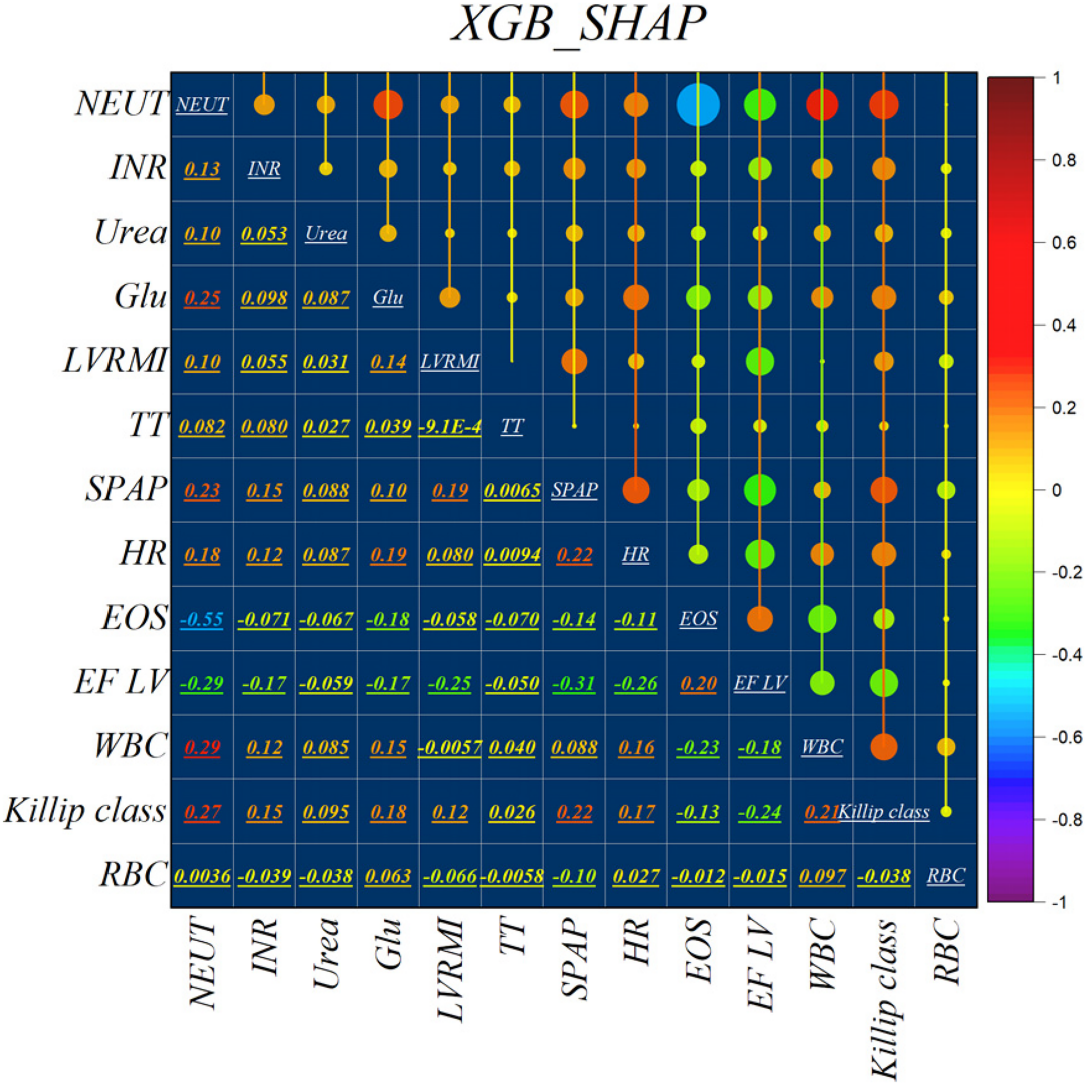
Feature selection and SHAP analysis for XGB.

Figure 2 illustrates the features identified by the SHAP method for the LGB model, while Figure 3 shows the selected features for the XGB model. In the LGB model, ten features were recognized as essential factors in modeling and forecasting, while the XGB model identified 13 features. After a comprehensive examination of the relationships, it becomes apparent that the correlation between systolic pulmonary arterial pressure and heart rate, along with the correlation between neutrophils and glucose, is direct. Conversely, the relationship between neutrophils and eosinophils shows an inverse trend.

#### 3.2.2 Recursive Feature Elimination (RFE)

The Recursive Feature Elimination (RFE) selection method (60) fundamentally operates through a recursive procedure wherein features are systematically ranked based on a specified measure of their significance.

A feature ranking criterion that performs well for individual features may not be suitable for assessing feature subsets effectively. Metrics such as Dj(i) or $(w_{i}{)}^{2}$ measure the impact of removing single features on the objective function but may struggle when removing multiple features simultaneously, which is crucial for obtaining a concise subset. To overcome this limitation, RFE employs an iterative approach to systematically remove the least relevant features in each iteration. RFE considers potential changes in feature importance across various feature subsets, particularly for highly correlated features. The order of feature elimination determines the final ranking, and the top *n* features are selected from this ranking for the feature selection process (61). Train the classifier, compute the ranking criterion for all features, and then remove the feature with the smallest ranking criterion.

When features are eliminated one by one, they are correspondingly ranked. However, the features ranked highest (eliminated last) may not necessarily be individually the most relevant. The optimal subset is determined by considering features collectively rather than individually. It is important to note that RFE does not affect correlation methods, as the ranking criterion is computed based solely on information from individual features. Table 2 reports the RFE ranking obtained for each feature in the dataset based on each base models and selected features.

**Table 2:** RFE ranking and selected features from the dataset based on each base model (scenario 3).

NO	Parameter	RFE ranking (LGB)	Selected features (LGB)	RFE ranking (XGB)	Selected features (XGB)
1	Sex	39		39	
2	Age	25		23	
3	Height	26		34	
4	Weight	28		30	
5	BMI	4	***✓***	38	
6	SBP	24		35	
7	DBP	34		16	
8	PBP	33		31	
9	HR	13		8	***✓***
10	Cr	15		17	
11	Killip class	30		7	***✓***
12	EF LV	14		14	
13	EDV	27		15	
14	ESV	20		13	
15	LVRWTI	1	***✓***	25	
16	LVRMI	2	***✓***	11	
17	SPAP	8		6	***✓***
18	La1	22		10	
19	La2	31		19	
20	Ra1	32		24	
21	Ra2	11		12	
22	WBC	9		20	
23	NEUT	6	***✓***	1	***✓***
24	LYM	17		26	
25	EOS	23		5	***✓***
26	RBC	18		22	
27	Hb	10		21	
28	PLT	16		36	
29	Glu	3	***✓***	9	
30	Urea	7		4	***✓***
31	PCT	29		29	
32	PI	19		32	
33	INR	12		2	***✓***
34	TT	5	***✓***	3	***✓***
35	APTT	21		37	
36	Form STEMI	37		28	
37	AFib	35		27	
38	Diabetes	38		33	
39	CKD	36		40	
40	aMI	40		41	
41	COPD	41		18	

**Figure 4:**
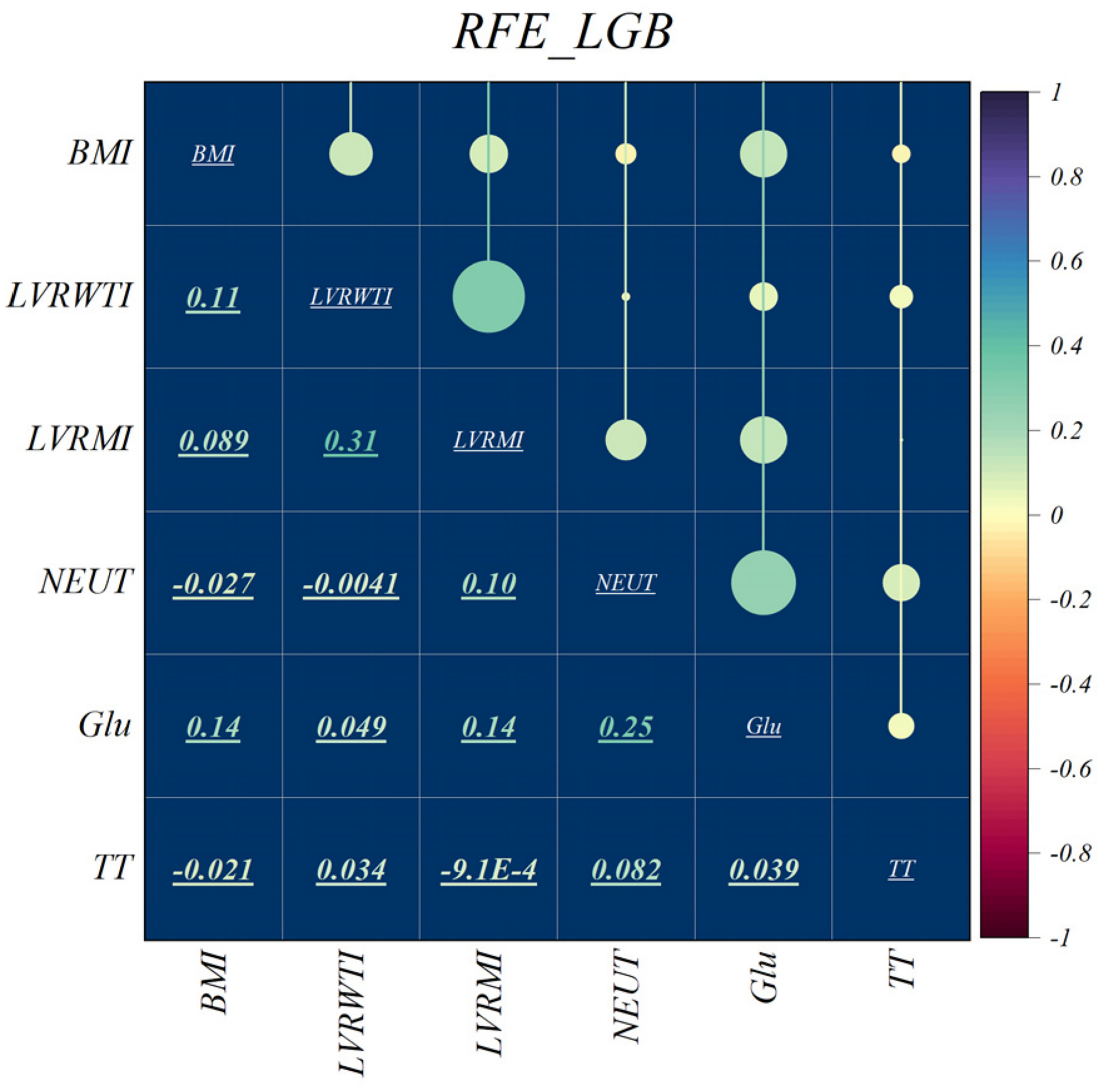
Feature selection and RFE analysis for LGB.

**Figure 5:**
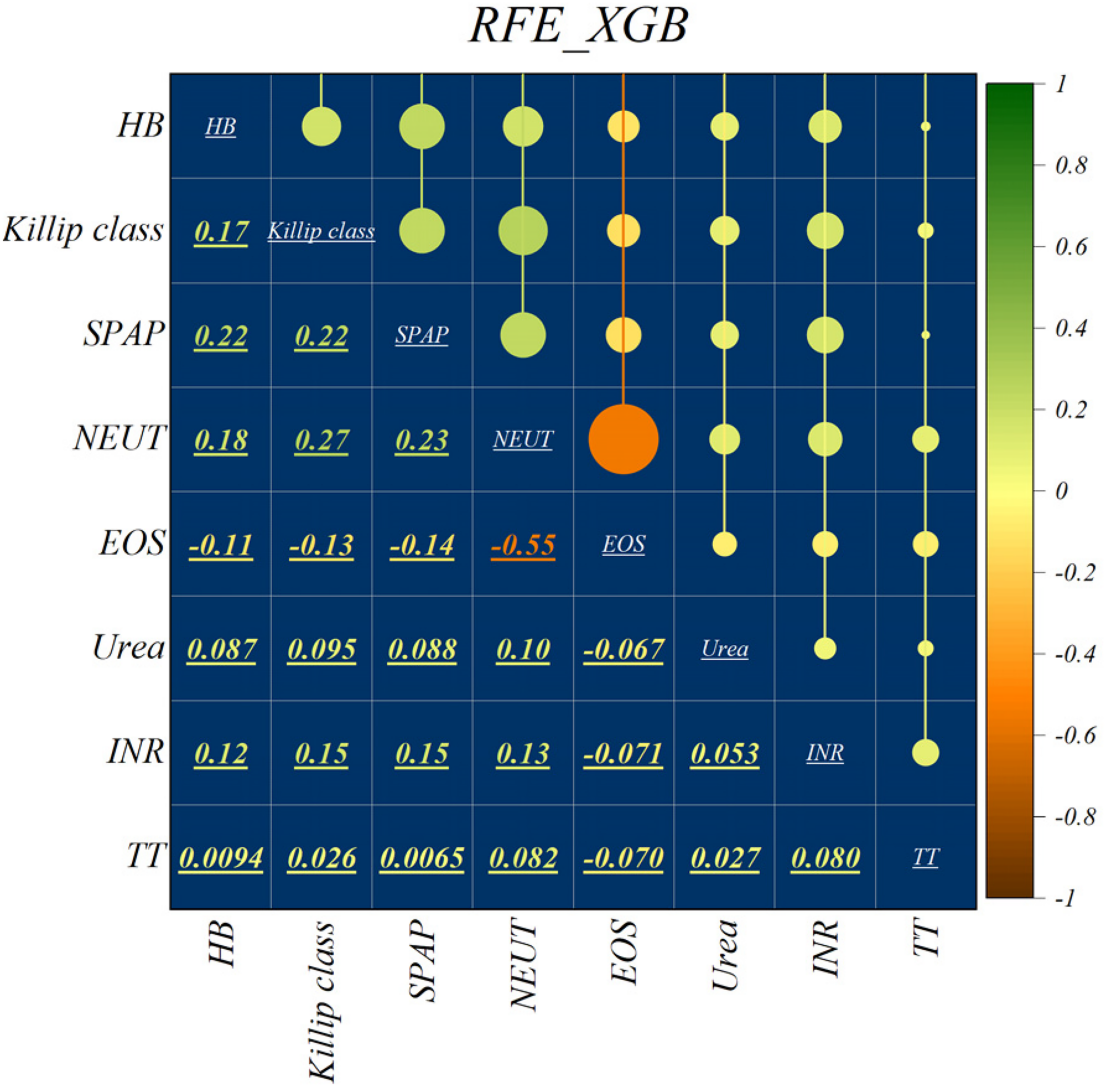
Feature selection and RFE analysis for XGB.

The features selected using RFE for the LGB and XGB models are visually depicted in Figures 4, 5, respectively. The selected features consist of 6 parameters for the LGB model and 8 for the XGB model. Upon scrutiny of the presented matrix, it becomes apparent that the left ventricular regional motion index and the left ventricular relative wall thickness index, both cardiovascular parameters, display a direct relationship with each other. Additionally, it is remarkable that neutrophils demonstrate a strong correlation with heart rate, systolic pulmonary arterial pressure, and Killip classification. Conversely, thrombin time shows no significant relationship with other selected parameters.

In this study, NEUT, TT, BUN, Glu, and SPAP were identified as key factors for the risk of IHM after PCI in patients with STEMI through the above-mentioned feature selection methods. NEUT play a central role in infection and inflammation, and their high levels in MI may indicate inflammatory processes associated with myocardial damage (62). Inflammation not only promotes atherosclerosis but may also lead to plaque rupture, increasing the risk of cardiac events (63). TT is an indicator for assessing the coagulation cascade, and its prolongation may suggest abnormal coagulation factor activity, increasing the risk of thrombosis after myocardial infarction (64). Additionally, prolonged TT may be associated with the use of anticoagulant drugs, which is common in the management of heart diseases. BUN reflects renal insufficiency in heart diseases, which may affect fluid and electrolyte balance, activate the renin-angiotensin-aldosterone system, leading to increased blood pressure and cardiac load, affecting cardiac function and clinical outcomes (65). High blood glucose is an independent risk factor for cardiovascular diseases, and chronic hyperglycemia promotes oxidative stress and inflammatory responses, leading to abnormal vascular endothelial function and accelerated atherosclerosis, exacerbating myocardial injury and the risk of cardiovascular events (66). Elevated SPAP is associated with changes in cardiac structure and function, and after myocardial infarction, it may indicate increased right ventricular load, leading to dysfunction, affecting the heart's pumping ability, increasing the risk of heart failure and death (67). These characteristics affect patient outcomes through various biological pathways, and a deeper understanding of these mechanisms can help better understand the disease development process and develop targeted treatment strategies.

## 4 Optimization methods

In this study, we combined four metaheuristic algorithms: the Augmented Grey Wolf Optimizer (AGWO), Bald Eagle Search Optimization (BES), Golden Jackal Optimizer (GJO), and Puma Optimizer (PO). These algorithms, each mimicking unique behaviors in nature, possess different search strategies that effectively avoid local optima and demonstrate efficient search capabilities and robustness in complex decision spaces. To optimize model performance, we employed grid search and cross-validation methods to fine-tune hyperparameters. Grid search systematically iterates through predefined hyperparameter values and evaluates each combination using cross-validation. Cross-validation divides the dataset into multiple subsets, using one subset as a test set and the rest as training sets, to assess the model's generalization ability. This study specifically utilized the Monte Carlo Cross-Validation (MCCV) method, which evaluates the performance of optimizers under different hyperparameter settings through random sampling to determine the optimal parameter combination, thereby maximizing the model's predictive accuracy.

### 4.1 Augmented Grey Wolf Optimizer (AGWO)

The AGWO algorithm emphasizes the search parameter (A), fluctuating the global Grey Wolf Optimization (GWO). It matches gray wolves’ hunting behavior, where a leader, α, directs the pack, supported by secondary wolves, β, aiding in decision-making. α represents the estimated outcomes targeted at resolving the research issue (68). The hunting process is categorized into four different sections as follows (69):

#### 4.1.1 Foraging for prey

Exploring prey locations is enabled through the divergence of search agents, a condition satisfied when |A| surpasses 1. Parameter *A*, essential in balancing exploration and exploitation, is primarily contingent upon parameter *a* as described in (Equation 19):

(19) $$\vec{a}=2-\cos(\mathrm{rand})\times t/\mathrm{Max}\_\mathrm{iter}$$

(20) $$\vec{A}=2-\vec{a}.\vec{r_{1}}-\vec{a}$$

(21) $$\vec{C}=2.\vec{r_{2}}$$

The parameter *a* randomly and nonlinearly transitions from 2 to 1 as the iteration (t) increases, while $r_{1}$ and $r_{2}$ represent consistently dispersed random vectors ranging between 0 and 1 (Equations 20, 21). This process continues until it reaches the maximum iteration.

#### 4.1.2 Surrounding the prey

The mathematical formulation relating to the encirclement of prey is described as follows (Equations 22, 23):

(22) $$\vec{D}=|\vec{C}.\vec{X_{\;pi}}-\vec{X_{i}}|$$

(23) $$\vec{X_{i+1}}=X_{\;pi}-\vec{A}.\vec{D}\;$$

X represents the vector indicating the location of the grey wolf, while $X_{p}$ signifies the vector demonstrating the location of the prey.

#### 4.1.3 Hunting and tracking

In the proposed AGWO algorithm (Algorithm 1), the strategy for hunting is determined exclusively by the parameters α and β, which are defined in (Equations 24–26).

(24) $$\vec{D_{a}}=|\vec{C_{1}}.\;\vec{X_{ai}}-\vec{X_{i}}|,\;\;\;\;\;\;\;\vec{D_{\beta }}=|\vec{C_{2}}.\vec{X_{\beta i}}-\vec{X_{i}}|$$

(25) $$\vec{X_{1}}=\vec{X_{ai}}-\vec{A_{1}}.\vec{D_{a}},\;\;\vec{X_{2}}=\vec{X_{\beta i}}-\vec{A_{2}}.\vec{D_{\beta }}$$

(26) $$\vec{X_{1+i}}=\vec{X_{1}}+\vec{X_{2}}/2$$

#### 4.1.4 Attacking the Pre

The coordinated efforts of search agents may aid in the process of preying on a target; this investigation is conducted when the magnitude of set *A* is less than one.

**Algorithm 1:** Pseudocode outlining the AGWO.

**Initialize** population of grey wolves randomly **Estimate** the fitness of each wolf in the population **Repeat** until stopping standards are met: Update α and β positions based on fitness Renew other wolves' positions based on α and β positions Apply search operator to explore new solutions Assess the fitness of new solutions Update α and β positions if necessary **Return** the best solution found

### 4.2 Bald Eagle Search Optimization (BES)

Alsattar et al. introduced the Bald Eagle Search (BES) algorithm, drawing inspiration from the discerning hunting strategy observed in bald eagles (70). This algorithm is arranged around three sequential phases reflective of the bald eagle's hunting behavior. Initially, the algorithm identifies spatial domains characterized by a delicate presence of potential targets. Subsequently, within these delineated spaces, extensive exploration is conducted to determine optimal solutions. Finally, similar to the decisive swooping action of the bald eagle, the algorithm strategically converges towards superior solutions (71). Through this emulation of the bald eagle's hunting strategy, the BES algorithm demonstrates a deliberate and efficient approach to optimization problem-solving (72).

#### 4.2.1 Space selection stage

During this phase, bald eagles strive to select a search area abundant with food, aiming for optimal conditions. Here is the mathematical representation of this stage (Equation 27):

(27) $$X_{\mathrm{new},i}=X_{\mathrm{best}}+\beta *r(X_{\mathrm{mean}}-X_{i})$$

β control's location changes; *r* is a random number between 0 and 1. $X_{\mathrm{new},i}$ is a new position, $X_{\mathrm{best}}$ is the best position found, $X_{\mathrm{mean}}$ is the average position of all eagles and $X_{i}$ is the current eagle's position.

#### 4.2.2 Searching-in-space stage

During this stage, the bald eagle conducts a methodical search across various directions within the designated space to locate potential prey. It strategically assesses optimal hunting positions and plans its swooping maneuvers accordingly. This stage can be succinctly described in mathematical terms as (Equations 28–34):

(28) $$X_{\mathrm{new},i}=X_{i}+f(i)*(X_{i}-X_{i+1})+g(i)*(X_{i}-X_{\mathrm{mean}})$$

(29) $$g(i)=\frac{\mathrm{gr}(i)}{\left(\max|\mathrm{gr}|\right)}$$

(30) $$f(i)=\frac{\mathrm{fr}(i)}{\left(\max|\mathrm{fr}|\right)}$$

(31) gr(i)=r(i).sin⁡(φ(i))

(32) fr(i)=r(i).cos⁡(φ(i))

(33) φ(i)=β.π.rand

(34) r(i)=φ(i)+S.rand

S quantifies the total number of search attempts, while β denotes the angle delineating the direction of the search. The term rand encompasses a numerical value within the inclusive range of 0 to 1.

#### 4.2.3 Swooping stage

In the final phase, each bald eagle begins a swinging motion from a superior location toward its predefined prey. The mathematical definition of this behavior in this phase is presented as follows (Equations 35–41):

(35) $$X_{\mathrm{new},i}=\mathrm{rand}.\;X_{\mathrm{best}}+g_{1}(i).(X_{i}-B_{1}.\;X_{\mathrm{mean}})+f_{1}(i).(X_{i}-B_{2}.\;X_{\mathrm{best}})$$

(36) $$g_{1}(i)=\frac{\mathrm{gr}(i)}{\left(\max|\mathrm{gr}|\right)}$$

(37) $$f_{1}(i)=\frac{\mathrm{fr}(i)}{\left(\max|\mathrm{fr}|\right)}$$

(38) gr(i)=r(i).sin⁡(φ(i))

(39) fr(i)=r(i).cos⁡(φ(i))

(40) φ(i)=β.π.rand

(41) r(i)=φ(i)

$$B_{1},\;B_{2}\epsilon [1,2]$$.

**Figure 6:**
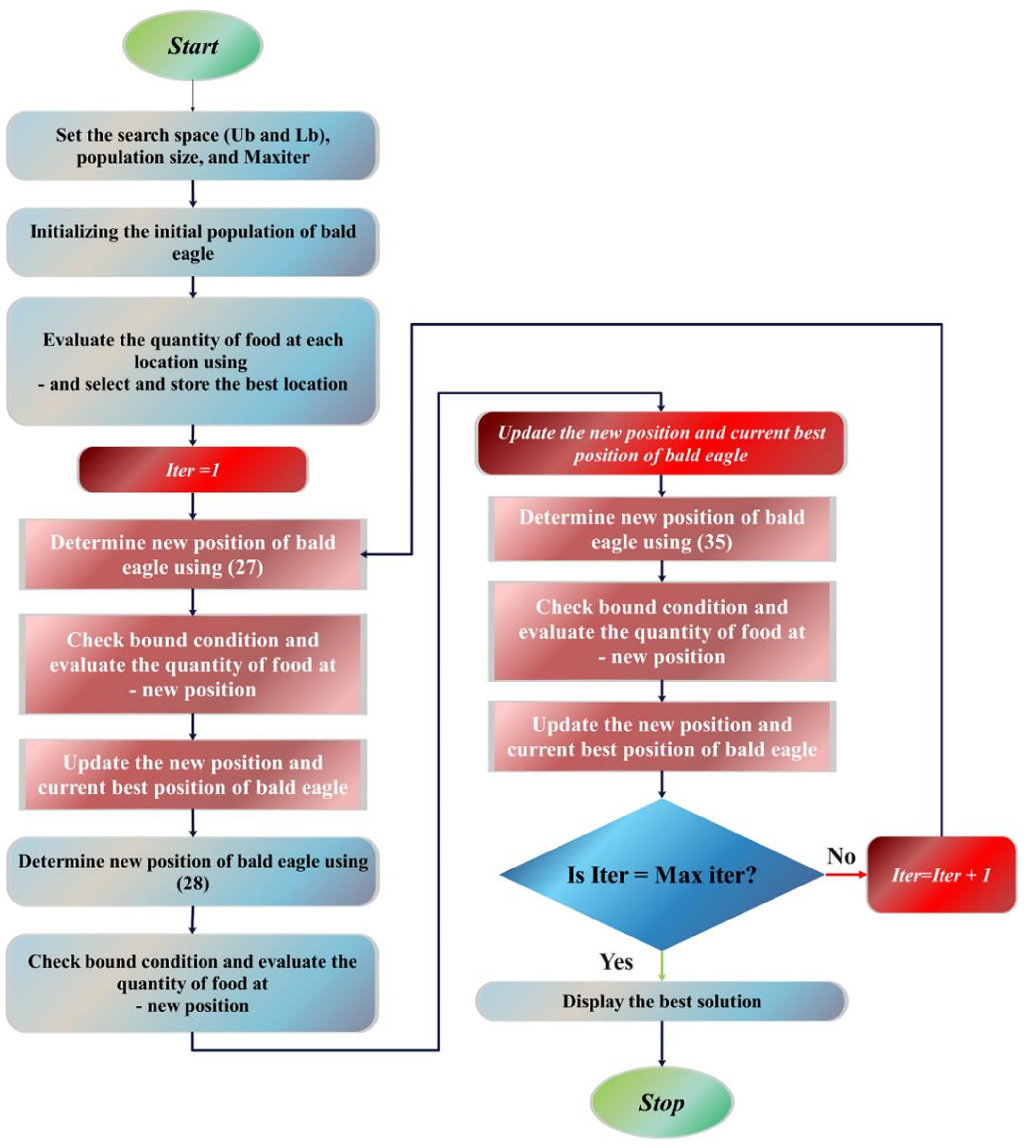
The flowchart of BES.

The comprehensive depiction of the BES algorithm is accessible through the subsequent pseudocode (Algorithm 2), and the flowchart of BES is illustrated in Figure 6.

**Algorithm 2:** Pseudocode outlining the Bald Eagle Search Optimization.

Randomly assign initial values: $X_{i}$ for *n* points. Determine the fitness values of the initial points: $f(X_{i})$ **While** (until the termination criteria are met) **Selecting Space** **For** (every individual *i* within the population) $X_{\mathrm{new}}=X_{\mathrm{best}}+\beta *r(X_{\mathrm{mean}}-X_{i})$ **If** $f(X_{\mathrm{new}})\;<f(X_{i})$ $X_{i}=X_{\mathrm{new}}$ **If** $f(X_{\mathrm{new}})<f(X_{\mathrm{best}})$ .$X_{\mathrm{best}}=X_{\mathrm{new}}$ **End If** **End If** **End For** **Searching in Space** **For** (each individual denoted as *i* within the population) $X_{new}=X_{i}+f(i)*(X_{i}-X_{i+1})+g(i)*(X_{i}-X_{mean})$ **If** $f(X_{\mathrm{new}})<f(X_{i})$ .$X_{i}=X_{\mathrm{new}}$ **If** $f(X_{\mathrm{new}})\;<f(X_{\mathrm{best}})$ .$X_{\mathrm{best}}=X_{\mathrm{new}}$ **End If** **End If** **End For** **Swooping and Descending** **For** (each memberi among the population) .$X_{new}=rand.\;X_{best}+g_{1}(i).(X_{i}-B_{1}.\;X_{mean})+f_{1}(i).(X_{i}-B_{2}.\;X_{best})$ **If** $f(X_{\mathrm{new}})<f(X_{i})$ .$X_{i}=X_{\mathrm{new}}$ **If** $f(X_{\mathrm{new}})<f(X_{\mathrm{best}})$ .$X_{\mathrm{best}}=X_{\mathrm{new}}$ **End If** **End If** **End For** Set k=k+1 **End While**

### 4.3 Golden Jackal Optimizer (GJO)

The Golden Jackal Optimizer (GJO) represents a recent advancement in swarm-based optimization methodologies strategically developed to optimize diverse engineering systems and processes (73). Drawing inspiration from the collaborative hunting tactics observed in golden jackals, the GJO includes three important subprocesses: *Prey Exploration*, *Surrounding*, and *Attacking* (74, 75). Within this section, the mathematical formulation of the GJO is clarified.

At the beginning of the optimization process, the generation of a set of prey location matrices is initiated, achieved via the randomization method described in (Equation 42):

(42) $$\left[\begin{array}{ccc} Y_{1,1} & \cdots & \begin{array}{cc} Y_{1,j} & \begin{array}{cc} \cdots & Y_{1,n} \end{array} \end{array}\\ Y_{2,1} & \cdots & \begin{array}{cc} Y_{2,j} & \begin{array}{cc} \cdots & Y_{2,n} \end{array} \end{array}\\ \begin{array}{c} \cdots \\ \vdots \\ \begin{array}{c} Y_{N-1,1}\\ Y_{N,1} \end{array} \end{array} & \begin{array}{c} \cdots \\ \vdots \\ \begin{array}{c} \cdots \\ \cdots \end{array} \end{array} & \begin{array}{c} \begin{array}{cc} \cdots \;\;\; & \begin{array}{cc} \cdots & \;\;\;\cdots \end{array} \end{array}\\ \begin{array}{cc} \vdots & \;\;\;\;\;\;\begin{array}{cc} \vdots \;\;\;\; & \vdots \end{array} \end{array}\\ \begin{array}{c} \begin{array}{cc} Y_{N-1,j} & \begin{array}{cc} \cdots & Y_{N-1,n} \end{array} \end{array}\\ \begin{array}{cc} Y_{N,j} & \begin{array}{cc} \cdots & Y_{N,n} \end{array} \end{array} \end{array} \end{array} \end{array}\right]$$

The method that the golden jackal hunts, where the *E* value is greater than 1, is illustrated numerically. *N* is the number of prey populations at this stage, and *n* is the total number of variables.

(43) $$Y_{1}(t)=Y_{M}(t)-E.|Y_{M}(t)-\mathrm{rl}.\mathrm{prey}(t)|$$

(44) $$Y_{2}(t)=Y_{\mathrm{FM}}(t)-E.|Y_{\mathrm{FM}}(t)-\mathrm{rl}.\mathrm{prey}(t)|$$

In the given equation, *t* represents the iteration number, $Y_{M}(t)$ and $Y_{\mathrm{FM}}(t)$ denote the positions of male and female golden jackals, respectively, while prey(t) represents the prey's position vector. The updated positions of the golden jackals are $Y_{1}(t)$ and $Y_{2}(t)$, respectively. The variable *E* signifies the prey's evading energy, calculated using a specific formula (Equations 45, 46):

(45) $$E=E_{1}.E_{0}$$

(46) $$E_{1}=c_{1}.\left(1-\left(\frac{t}{T}\right)\right)$$

The equation assesses the ability of prey to avoid predators, considering several aspects. Firstly, a random value within the range of −1 to 1, denoted as $E_{0}$, represents the prey's starting energy level. The parameter *T* signifies the maximum number of iterations, while $c_{1}$ is a constant value typically set to 1.5. $E_{1}\;$ indicates how quickly the prey's energy decreases over time.(Equations 47, 48) apply the distance between the golden jackal and the prey, expressed as $\left|Y_{M}(t)-\mathrm{rl}.\mathrm{prey}(t)\right|$, where rl denotes a vector of random numbers resulting from the *Levy* flight function.

(47) rl=0.05.LF(y)

(48) $$\begin{array}{rl} \mathrm{LF}(y)= & \;0.01\times (\mu \times \sigma )/(\left|v^{\left(1/\beta \right)}\right|),\;\;\;\;\;\;\;\;\;\;\;\;\\ \sigma = & \left\{\frac{\Gamma (1+\beta )\times \sin(\pi \beta /2)}{\Gamma \left(\frac{1+\beta }{2}\right)\times \beta \times (2^{\beta -1})}\right\} \end{array}$$

The calculation uses random values for *u* and *v* that fall between 0 and 1, and it also includes a constant *b* that is often set to 1.5 by default.

(49) $$Y(t+1)=\frac{Y_{1}(t)+Y_{2}(t)}{2}$$

The formula calculates the prey's updated location, Y(t+1), based on the positions of the male and female golden jackals.

The reduced capability of the prey to evade emerges when it faces violence from the golden jackals. This mathematical expression illustrates a decline in evading energy when |E| is less than or equal to 1.

(50) $$Y_{1}(t)=Y_{M}(t)-E.|\mathrm{rl}.Y_{M}(t)-\mathrm{prey}(t)|$$

(51) $$Y_{2}(t)=Y_{\mathrm{FM}}(t)-E.|\mathrm{rl}.Y_{\mathrm{FM}}(t)-\mathrm{rl}.\mathrm{prey}(t)|$$

**Figure 7:**
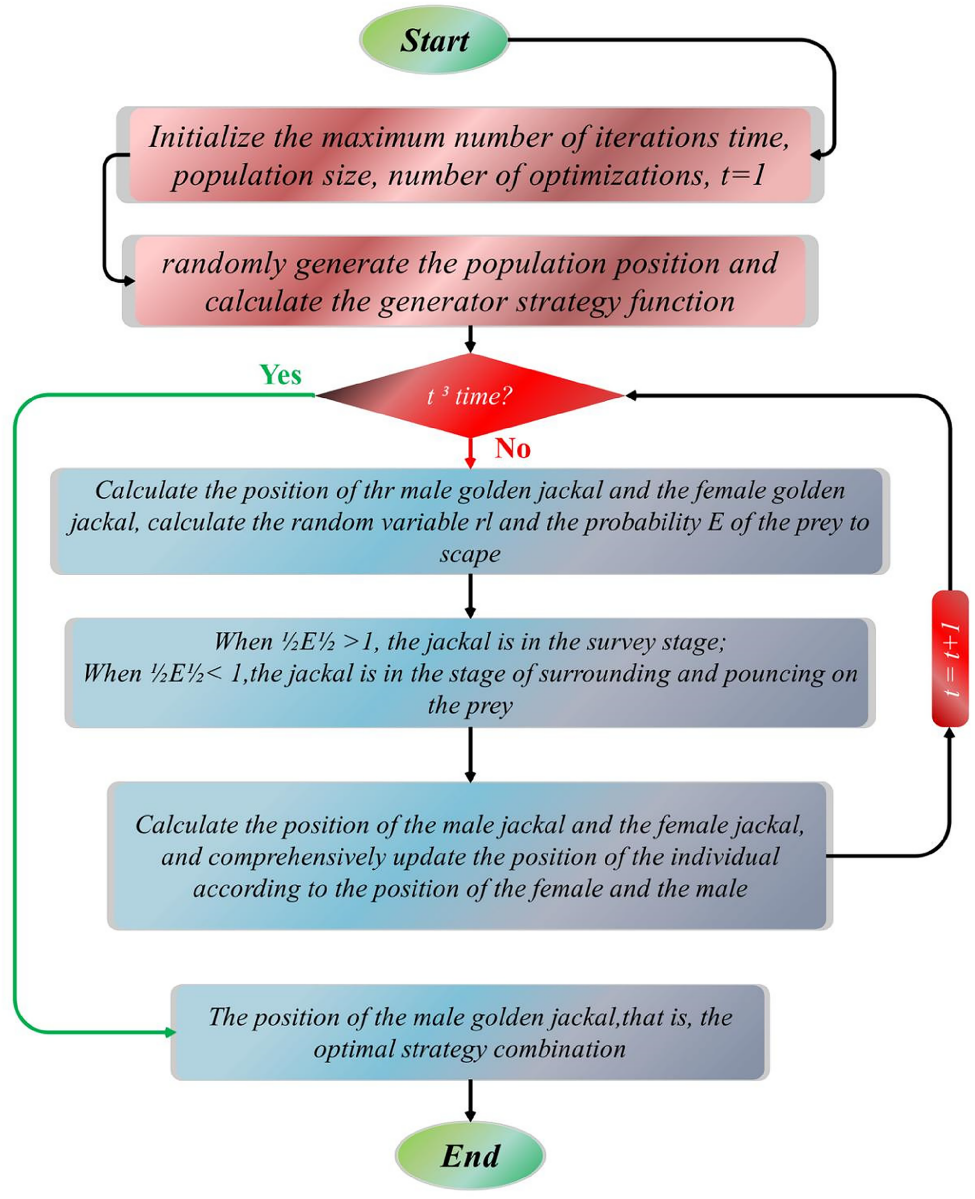
The flowchart of GJO.

The comprehensive depiction of the GJO algorithm is outlined in the pseudocode provided below (Algorithm 3) and Figure 7 illustrates the flowchart of GJO.

**Algorithm 3:** Pseudocode delineation of the Golden Jackal Optimizer.

**Inputs**: The population size *N* and maximum number of iterations T **Outputs**: The position of the prey and its corresponding fitness value Initiate the random population of prey denoted as $Y_{i}$ for i=1,2,...,N ** While** (t<T) ** **Compute the fitness values of the prey ** **$Y_{1}$ = determine the best position of the male jackal among the prey individuals ** **$Y_{2}$ = identify the second-best position of the female jackal among the prey individuals ** For** (each member of the prey population) ** **Modify the evading energy E in accordance with (Equations 47, 49) ** **Adjust the variable rl by applying (Equations 49, 50) ** If** (|E|≤1) (Exploration Stage) ** **Refine the prey's location in space via the use of (Equations 43, 44, 49). ** If** (|E|>1) (Exploitation Stage) ** **Adjust the prey's position based on (Equations 49–51). ** End For** .** **t=t+1 ** End While** Return $Y_{1}$

### 4.4 Puma optimizer (PO)

The Puma algorithm was subjected to review by Abdollah Zadeh et al. (76), and its description is as follows:

#### 4.4.1 Inspiration

The Puma, also called cougar or mountain lion, is a large American feline found across a vast range from the Andes to Canada. It is known for its adaptability, nocturnal nature, and ambush hunting style, preying on deer, rodents, and occasionally domestic animals (77–79). Pumas prefer dense scrub and rocky habitats, establish large territories, and display typical territorial behavior (80). They typically capture large prey every two weeks, relocating it for feeding over several days. Pumas are solitary, except for mothers and cubs, and rarely encounter each other except to share prey or in small communities centered around a dominant male's territory (81).

#### 4.4.2 Mathematical representation

This section presents the PO algorithm, which draws inspiration from the hunting behaviors of pumas. Different from conventional meta-heuristic optimizers, PO introduces a unique mechanism for transitioning between the exploration and exploitation phases. It conceptualizes the best solution as a male puma and views the entire optimization space as a puma's territory, with other solutions representing female pumas. Purposeful and intelligent phase selection guides solutions through exploration or exploitation in each iteration. Drawing from puma behavior, diverse optimization approaches are employed in each phase, enhancing the algorithm's efficiency.

##### 4.4.2.1 Puma-inspired intelligence (phase transition mechanism)

The algorithm, inspired by puma behavior, features an exploitation phase for revisiting known hunting grounds and an exploration phase for discovering new territories. It incorporates a sophisticated mechanism resembling an advanced hyper-heuristic algorithm, integrating diversity and intensification components for scoring. The phase transition section adopts two approaches inspired by puma intelligence: inexperienced pumas explore new territories while targeting promising areas for ambush.

###### 4.4.2.1.1 Inexperienced phase

In its early stages, the puma lacks experience and often engages in exploration activities simultaneously due to its unfamiliarity with its environment and lack of awareness of hunting locations within its territory. Conversely, it seeks hunting opportunities in favorable areas. In the Puma algorithm, during the initial three iterations, both exploration and exploitation operations are carried out concurrently until initialization is completed in the phase transition phase. In this section, as the exploitation and exploration phases are selected in each iteration, only two functions ($f_{1}$ and $f_{2}$) are applied and calculated using (Equations 52–55):

(52) $$f_{1\mathrm{Explor}}=PF_{1}.\left(\frac{{\mathrm{Seq}}_{\mathrm{costExplore}}^{1}}{\mathrm{Se}q_{\mathrm{Time}}}\right)$$

(53) $$f_{1\mathrm{Exploit}}=PF_{1}.\left(\frac{{\mathrm{Seq}}_{\mathrm{costExploit}}^{1}}{\mathrm{Se}q_{\mathrm{Time}}}\right)$$

(54) $$f_{2\mathrm{Explor}}=PF_{2}.\left(\frac{{\mathrm{Seq}}_{\mathrm{costExplore}}^{1}+{\mathrm{Seq}}_{\mathrm{costExplore}}^{2}+{\mathrm{Seq}}_{\mathrm{costExplore}}^{3}}{{\mathrm{Seq}}_{\mathrm{Time}}^{1}+{\mathrm{Seq}}_{\mathrm{Time}}^{2}+{\mathrm{Seq}}_{\mathrm{Time}}^{3}}\right)$$

(55) $$f_{2\mathrm{Exploit}}=PF_{2}.\left(\frac{{\mathrm{Seq}}_{\mathrm{costExploit}}^{1}+{\mathrm{Seq}}_{\mathrm{costExploit}}^{2}+{\mathrm{Seq}}_{\mathrm{costExploit}}^{3}}{{\mathrm{Seq}}_{\mathrm{Time}}^{1}+{\mathrm{Seq}}_{\mathrm{Time}}^{2}+{\mathrm{Seq}}_{\mathrm{Time}}^{3}}\right)$$

The values of $\mathrm{Se}q_{\mathrm{cost}}$, associated with both exploitation and exploration phases, are determined using (Equations 52–55), while $\mathrm{Se}q_{\mathrm{Time}}$ remains constant at 1. $PF_{1}$ and $PF_{2}$, parameters with predetermined values, are used to prioritize the functions $f_{1}$ and $f_{2}$ during the optimization process.

(56) $${\mathrm{Seq}}_{\mathrm{CostExplore}}^{1}=|{\mathrm{Cost}}_{\mathrm{Best}}^{\mathrm{Initial}}-{\mathrm{Cost}}_{\mathrm{Exlore}}^{1}|$$

(57) $${\mathrm{Seq}}_{\mathrm{CostExplore}}^{2}=|{\mathrm{Cost}}_{\mathrm{Explore}}^{2}-{\mathrm{Cost}}_{\mathrm{Exlore}}^{1}|$$

(58) $${\mathrm{Seq}}_{\mathrm{CostExplore}}^{3}=|{\mathrm{Cost}}_{\mathrm{Explore}}^{3}-{\mathrm{Cost}}_{\mathrm{Exlore}}^{2}|$$

(59) $${\mathrm{Seq}}_{\mathrm{CostExploit}}^{1}=|{\mathrm{Cost}}_{\mathrm{Best}}^{\mathrm{Initial}}-{\mathrm{Cost}}_{\mathrm{Exloit}}^{1}|$$

(60) $${\mathrm{Seq}}_{\mathrm{CostExploit}}^{2}=|{\mathrm{Cost}}_{\mathrm{Exploit}}^{2}-{\mathrm{Cost}}_{\mathrm{Exloit}}^{1}|$$

(61) $${\mathrm{Seq}}_{\mathrm{CostExploit}}^{3}=|{\mathrm{Cost}}_{\mathrm{Exploit}}^{3}-{\mathrm{Cost}}_{\mathrm{Exloit}}^{2}|$$

In Equations 56, 61, the term ${\mathrm{Cost}}_{\mathrm{Best}}^{\mathrm{Initial}}$ represents the cost of the initial optimal solution generated during the initialization phase. Additionally, six variables, namely ${\mathrm{Cost}}_{\mathrm{Exlore}}^{1}$, ${\mathrm{Cost}}_{\mathrm{Exlore}}^{2}$, ${\mathrm{Cost}}_{\mathrm{Exlore}}^{3}$, ${\mathrm{Cost}}_{\mathrm{Exloit}}^{1}$, ${\mathrm{Cost}}_{\mathrm{Exloit}}^{2}$, and ${\mathrm{Cost}}_{\mathrm{Exloit}}^{3}$, denote the costs associated with the best solutions obtained from the exploitation and exploration phases across three repetitions (Equations 57–60).

After evaluating the functions $f_{1}$ and $f_{2}$ following the third iteration, a decision is made to exclusively pursue either exploration or exploitation phases. The positive experiences of other Pumas influence this choice. To determine which phase to prioritize, the coordinates of both the exploitation and exploration points are computed by applying (Equations 62, 63):

(62) $$\mathrm{Scor}e_{\mathrm{Explore}}=(PF_{1}.f_{1\mathrm{Explor}})+(PF_{2}.f_{2\mathrm{Explor}})$$

(63) $$\mathrm{Scor}e_{\mathrm{Exploit}}=(PF_{1}.f_{1\mathrm{Exploit}})+(PF_{2}.f_{2\mathrm{Exploit}})$$

After computing $\mathrm{Scor}e_{\mathrm{Explore}}$ and $\mathrm{Scor}e_{\mathrm{Exploit}}$ using (Equations 62, 63), the system determines whether to proceed with the exploration or exploitation phase based on their values. If $\mathrm{Scor}e_{\mathrm{Exploit}}\;\geq \mathrm{Scor}e_{\mathrm{Explore}}$, the exploitation stage is entered; otherwise, the exploration step is chosen. However, a serious consideration arises at the end of the third iteration: each step independently generates solutions exceeding the total population size. To address this, the total cost of solutions from both phases is calculated at the end of the third iteration. Only the best solutions from the entire pool are retained, ensuring that the population size remains constant by replacing the current solutions.

###### 4.4.2.1.2 Experienced and Skilled phase

After three generational iterations, the Pumas acquire a satisfactory level of experience to opt for a singular optimization phase for subsequent iterations. Within this phase, three distinct scoring functions, namely $f_{1}$, $f_{2}$, and $f_{3}$, are applied. The main function, $f_{1}$, prioritizes either the exploration or exploitation phase based on their comparative performance, with a particular emphasis on the exploration phase. This function is determined using (Equation 52).

(64) $$f_{1t}^{\mathrm{exploit}}=PF_{1}.\left|\frac{{\mathrm{Cost}}_{\mathrm{old}}^{\mathrm{exploit}}-{\mathrm{Cost}}_{\mathrm{new}}^{\mathrm{exploit}}}{T_{t}^{\mathrm{exploit}}}\right|$$

(65) $$f_{1t}^{\mathrm{exploit}}=PF_{1}.\left|\frac{{\mathrm{Cost}}_{\mathrm{old}}^{\mathrm{explore}}-{\mathrm{Cost}}_{\mathrm{new}}^{\mathrm{explore}}}{T_{t}^{\mathrm{explore}}}\right|$$

Equations 64, 65 define $f_{1t}^{\mathrm{exploit}}$ and $f_{1t}^{\mathrm{exploit}}$ for the exploitation and exploration phases at iteration *t*. ${\mathrm{Cost}}_{\mathrm{old}}^{\mathrm{exploit}}$ and ${\mathrm{Cost}}_{\mathrm{new}}^{\mathrm{explore}}$ are costs before and after improving the current selection, while $T_{t}^{\mathrm{explore}}$ and $T_{t}^{\mathrm{exploit}}$ indicate unselected iterations. $PF_{1}$, set between 0 and 1, determines the importance of the first function: advanced values prioritize it.

The second function gives preference to the phase that outperforms the other, focusing on resonance. It assesses good performances sequentially, aiding in the selection of the exploitation phase. (Equations 66, 67) are employed to calculate this function.

(66) $$\begin{array}{rl} & f_{2t}^{\mathrm{exploit}}=PF_{2}.\\ & \left|\frac{\left({\mathrm{Cost}}_{\mathrm{old}.1}^{\mathrm{exploit}}-{\mathrm{Cost}}_{\mathrm{new},1}^{\mathrm{exploit}})+({\mathrm{Cost}}_{\mathrm{old}.2}^{\mathrm{exploit}}-{\mathrm{Cost}}_{\mathrm{new},2}^{\mathrm{exploit}})+({\mathrm{Cost}}_{\mathrm{old}.3}^{\mathrm{exploit}}-{\mathrm{Cost}}_{\mathrm{new},3}^{\mathrm{exploit}}\right)}{T_{t.1}^{\mathrm{exploit}}+T_{t.2}^{\mathrm{exploit}}+T_{t.3}^{\mathrm{exploit}}}\right| \end{array}$$

(67) $$\begin{array}{rl} & f_{2t}^{\mathrm{explore}}=PF_{2}.\\ & \left|\frac{\left({\mathrm{Cost}}_{\mathrm{old}.1}^{\mathrm{explore}}-{\mathrm{Cost}}_{\mathrm{new},1}^{\mathrm{explore}})+({\mathrm{Cost}}_{\mathrm{old}.2}^{\mathrm{explore}}-{\mathrm{Cost}}_{\mathrm{new},2}^{\mathrm{explore}})+({\mathrm{Cost}}_{\mathrm{old}.3}^{\mathrm{explore}}-{\mathrm{Cost}}_{\mathrm{new},3}^{\mathrm{explore}}\right)}{T_{t.1}^{\mathrm{explore}}+T_{t.2}^{\mathrm{explore}}+T_{t.3}^{\mathrm{explore}\;}}\right| \end{array}$$

Equations 66, 67 introduce functions for exploration and exploitation in an optimization process, with costs representing solution performance. Updates to solution costs are tracked across current and past selections. Iteration counts capture unselected iterations between selections. The parameter $PF_{2}$ influences the effectiveness of the exploration-exploitation balance. Overall, these elements form a framework for optimizing strategies.

The third function in the selection mechanism emphasizes diversity by increasing in value when its priority rises and decreasing when it declines. It ensures that less frequently selected phases still have a chance to be chosen, preventing the algorithm from getting trapped in local optima. This function is depicted in (Equations 68, 69).

(68) $$f_{3t}^{\mathrm{exploit}}=\{\begin{array}{c} \mathrm{if}\;\mathrm{selected},\;f_{3t}^{\mathrm{exploit}}=0\;\\ \mathrm{otherwise},\;\;\;f_{3t}^{\mathrm{exploit}}+PF_{3} \end{array}$$

(69) $$f_{3t}^{\mathrm{explore}}=\{\begin{array}{c} \mathrm{if}\;\mathrm{selected},\;f_{3t}^{\mathrm{explore}}=0\;\\ \mathrm{otherwise},\;\;\;f_{3t}^{\mathrm{explore}}+PF_{3} \end{array}$$

Equations 68, 69 define functions $f_{3t}^{\mathrm{exploit}}$ and $f_{3t}^{\mathrm{explore}}$ separately, representing the third function in exploitation and exploration stages over iterations signified by *t*. (Equation 54) specifies that if a stage is not chosen, the value of its corresponding third function increases by a parameter $PF_{3}$ in each iteration; otherwise, it is set to zero. $PF_{3}$ is a user-adjustable parameter ranging between 0 and 1, determining the likelihood of selecting a stage. A higher $PF_{3}$ increases the chances of selecting the stage with a lower score and vice versa. (Equations 70, 71) compute the cost associated with changing stages.

(70) $$F_{t}^{\mathrm{exploit}}=(\alpha_{t}^{\mathrm{exploit}}.(\;f_{1t}^{\mathrm{exploit}}))+(\alpha_{t}^{\mathrm{exploit}}.(\;f_{2t}^{\mathrm{exploit}}))+(\delta_{t}^{\mathrm{exploit}}.(lc.\;f_{3t}^{\mathrm{exploit}}))$$

(71) $$F_{t}^{\mathrm{explore}}=(\alpha_{t}^{\mathrm{explore}}.(\;f_{1t}^{\mathrm{explore}}))+(\alpha_{t}^{\mathrm{explore}}.(\;f_{2t}^{\mathrm{explore}}))+(\delta_{t}^{\mathrm{explore}}.(\mathrm{lc}.\;f_{3t}^{\mathrm{explore}}))$$

(72) $$\begin{array}{rl} & c=\{{\left\{\left|\mathrm{Cos}t_{\mathrm{old}}-\mathrm{Cos}t_{\mathrm{new}}\right|\right\}}^{\mathrm{exploitation}},{\left\{\left|\mathrm{Cos}t_{\mathrm{old}}-\mathrm{Cos}t_{\mathrm{new}}\right|\right\}}^{\mathrm{exploration}}\},\;\\ \; & 0\notin lc \end{array}$$

(73) $$\begin{array}{rl} & \alpha_{t}^{\mathrm{explore},\mathrm{exploit}}=\\ & \{\begin{array}{c} \mathrm{if}\;F^{\mathrm{exploit}}>F^{\mathrm{explore}},\alpha^{\mathrm{exploit}}=0.99,\alpha^{\mathrm{explore}}=\alpha^{\mathrm{explore}}-0.01,0.01\\ \mathrm{otherwise},\alpha^{\mathrm{explore}}=0.99,\alpha^{\mathrm{exploit}}=\;\alpha^{\mathrm{exploit}}-0.01,0.01 \end{array} \end{array}$$

(74) $$\delta_{t}^{\mathrm{exploit}}=1-\alpha_{t}^{\mathrm{exploit}}$$

(75) $$\delta_{t}^{\mathrm{explore}}=1-\alpha_{t}^{\mathrm{explore}}$$

Equations 70, 71 determine final costs for exploitation and exploration phases, with parameters *a* and *d* varying based on phase results, prioritizing diversity. (Equation 73) penalizes parameter *a* of the phase with higher cost, adjusting it linearly by 0.01. This approach, as discussed in (82), relies on lc, representing non-zero cost differences between exploitation and exploration phases (Equation 72).

##### 4.4.2.2 Exploration

In the exploration phase, inspired by the behavior of pumas searching for food, a random search is conducted within the territory. Pumas either explore new areas or approach other pumas to potentially share prey. Initially, the entire population is sorted in ascending order, and then each puma refines its solutions using (Equations 74, 75).

(76) $$\begin{array}{c} \mathrm{If}\;\mathrm{ran}d_{1}>0.5,\;\;Z_{i,G}=R_{\mathrm{Dim}}*(\mathrm{Ub}-\mathrm{LB})+\mathrm{LB}\;\;\\ \mathrm{Otherwise},\;Z_{i,G}=X_{a,G}+G.(X_{a,G}-X_{b,G})+G.((\left(X_{a,G}-X_{b,G})-(X_{c,G}-X_{d,G}\right))\;\\ \;\;\;\;\;\;+\;(\left(X_{c,G}-X_{d,G})-(X_{e,G}-X_{\;f,G}\right)))\; \end{array}$$

(77) $$G=2.\mathrm{ran}d_{2}-1$$

Equations 76, 77 involves randomly generating numbers within specified bounds and dimensions for problem-solving. Depending on certain conditions, one of two equations is selected to produce a new solution. This solution is then used to improve the current solution (Equations 78–81).

(78) $$X_{\mathrm{new}}=\{\begin{array}{c} Z_{i.G},\;\mathrm{if}\;j=j_{\mathrm{rand}}\;\mathrm{or}\;\mathrm{ran}d_{3}\leq U\\ X_{a,G},\;\mathrm{otherwise} \end{array}$$

(79) NC=1−U

(80) $$p=\frac{\mathrm{NC}}{N_{\mathrm{pop}}}$$

(81) $$\mathrm{if}\;\mathrm{Cost}\;X_{\mathrm{new}}<\;\mathrm{Cost}\;X_{i},\;U=U+P$$

##### 4.4.2.3 Exploitation

In the exploitation stage, the PO algorithm employs two operators inspired by puma behaviors: ambush hunting and sprinting. Pumas, in nature, typically ambush prey from concealed positions or chase them down in open spaces. (Equation 82) simulates the behavior of chasing prey, reflecting one of these hunting strategies.

(82) $$\begin{array}{rl} & X_{\mathrm{new}}=\\ & \left\{ \begin{array}{c} \mathrm{if}\;\mathrm{ran}d_{4}\geq 0.5,\;X_{\mathrm{new}}=\frac{\left(\frac{\mathrm{mean}(\mathrm{So}l_{\mathrm{total}})}{N_{\mathrm{pop}}}\right).X_{1}^{r}-{\left(-1\right)}^{\beta }\times X_{i}}{1+(\alpha .\mathrm{ran}d_{5})}\;\\ \mathrm{otherwise},\;\mathrm{if}\;\mathrm{ran}d_{6}\geq L,\;X_{\mathrm{new}}=\mathrm{Pum}a_{\mathrm{male}}+(2.\mathrm{ran}d_{7}).\exp(\mathrm{ran}d_{1}).X_{2}^{r}-X_{i}\;\\ \mathrm{otherwise},X_{\mathrm{new}}=(2\times \mathrm{ran}d_{8})\times \;\frac{F_{1}.R.X(i)+F_{2}.(1-R).\mathrm{Pum}a_{\mathrm{male}})}{\left(2.\mathrm{ran}d_{9}-1+\mathrm{rand}n_{2}\right)}-\mathrm{Pum}a_{\mathrm{male}}\; \end{array}\right. \end{array}$$

Equation 82 in the PO algorithm embodies two strategies inspired by puma behaviors: fast running and ambush hunting. During exploitation, if a randomly generated number exceeds 0.5, the fast-running strategy is applied; otherwise, the ambush strategy is chosen. These strategies involve different movements towards prey, with various parameters and random factors influencing the process.

The Puma optimizer stands out for its higher implementation complexity compared to other optimizers due to its multiple phases and operations involved in creating intelligent systems. In each iteration, the cost function is applied only once for each search agent, ensuring acceptable computational complexity, as detailed in the relevant section.

### 4.5 Hybrid models’ development

AGWO, BES, GJO, and PO optimizers integrated with base models to supplement the efficacy of the selected models. As presented in Tables 3, 4, the fine tunned hyperparameters in the hybridization process for LGBC and XGBC are reported. For instance, the hyperparameters n_estimators, max_depth, and learning_rate are crucial for optimizing ensemble methods like Gradient Boosting Machines. n_estimators define the number of trees in the ensemble, with more trees generally improving performance but increasing computational cost and overfitting risk. max_depth limits the depth of each tree, balancing the ability to capture complex patterns with the risk of overfitting; deeper trees can capture more details but may overfit, while shallower trees might underfit. learning_rate, specific to boosting algorithms, scales the contribution of each tree, with lower rates enhancing robustness and preventing overfitting but requiring more iterations.

**Table 3:** The results of hyperparameters tunning in LGBC-based hybrid models development.

Prediction scenarios	Model	Hyperparameter				
		num_leaves	max_depth	learning_rate	n_estimators	max_bin
Scenario 1	LGAG	321	285	0.168	317	681,000
	LGBE	131	148	0.361	119	289,000
	LGGJ	792	45	0.747	796	844,000
	LGPO	613	999	0.504	999	1,000
Scenario 2	LGAG	654	213	0.566	813	685,100
	LGBE	999	648	0.736	889	753,000
	LGGJ	981	539	0.746	822	788,114
	LGPO	274	921	0.497	867	66,000
Scenario 3	LGAG	213	286	0.126	681	385,140
	LGBE	140	411	0.651	214	293,000
	LGGJ	442	845	0.740	226	922,419
	LGPO	299	633	0.771	77	253,000

**Table 4:** The results of hyperparameters tunning in XGBC-based hybrid models development.

Prediction scenarios	Model	Hyperparameter						
		n_estimators	max_depth	learning_rate	colsample_bytree	subsample	reg_alpha	reg_lambda
Scenario 1	XGAG	581	48	0.237	0.371	0.382	0.14	0.641
	XGBE	628	134	0.196	0.657	0.658	0.007	0.189
	XGGJ	414	282	0.474	0.902	0.894	0.334	0.005
	XGPO	341	999	0.927	0.999	0.873	0.001	0.999
Scenario 2	XGAG	168	34	0.2781	0.391	0.617	0.236	0.468
	XGBE	345	315	0.16	0.152	0.294	0.067	0.065
	XGGJ	478	167	0.933	0.465	0.590	0.236	0.005
	XGPO	999	234	0.99	0.269	0.999	0.999	0.001
Scenario 3	XGAG	691	31	0.017	0.681	0.914	0.642	0.260
	XGBE	333	371	0.324	0.578	0.484	0.143	0.143
	XGGJ	201	393	0.208	0.232	0.393	0.635	0.005
	XGPO	394	595	0.614	0.488	0.667	0.001	0.072

**Figure 8:**
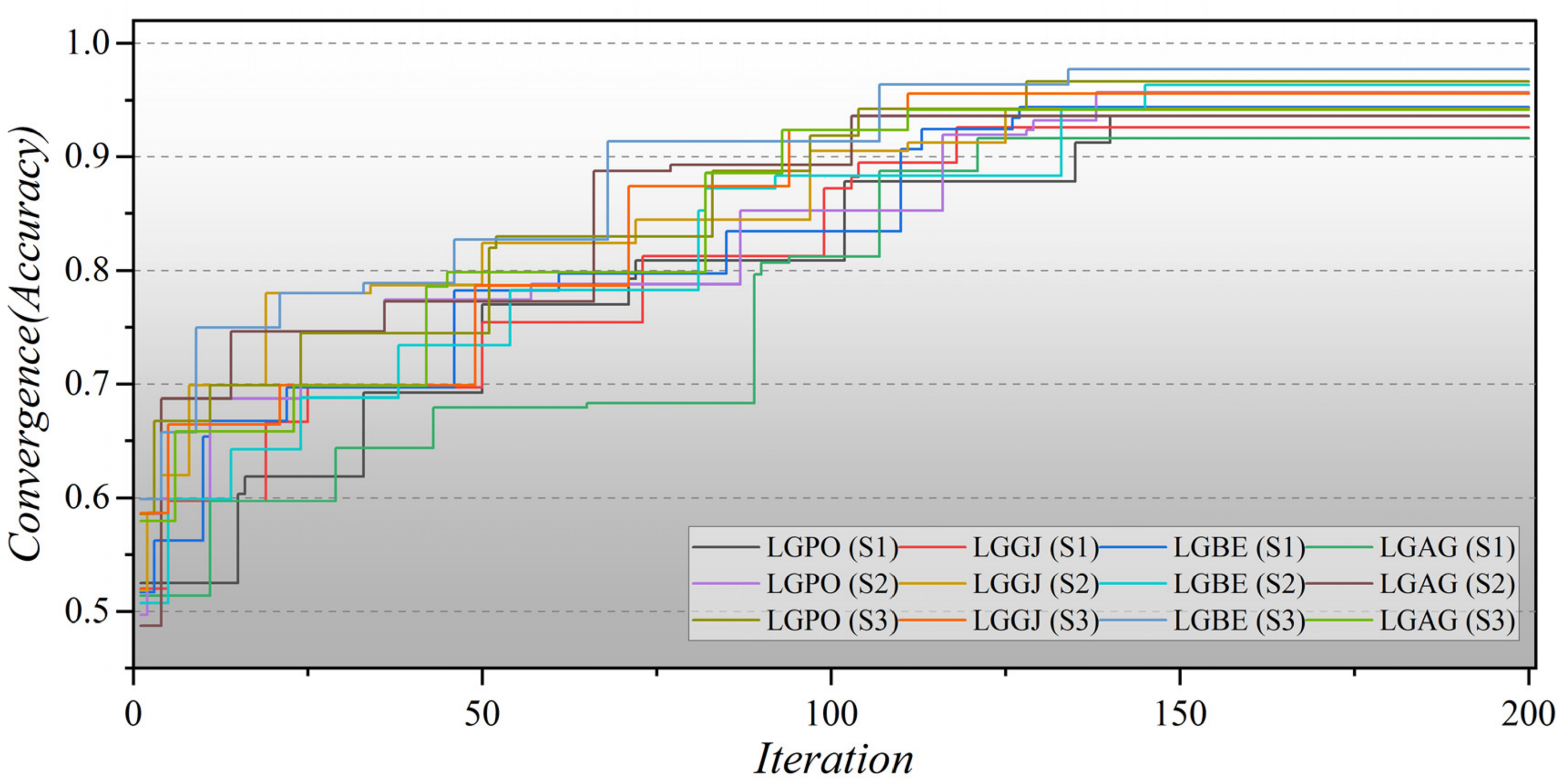
The convergence plot of LGB-based hybrid models in all three scenarios.

**Figure 9:**
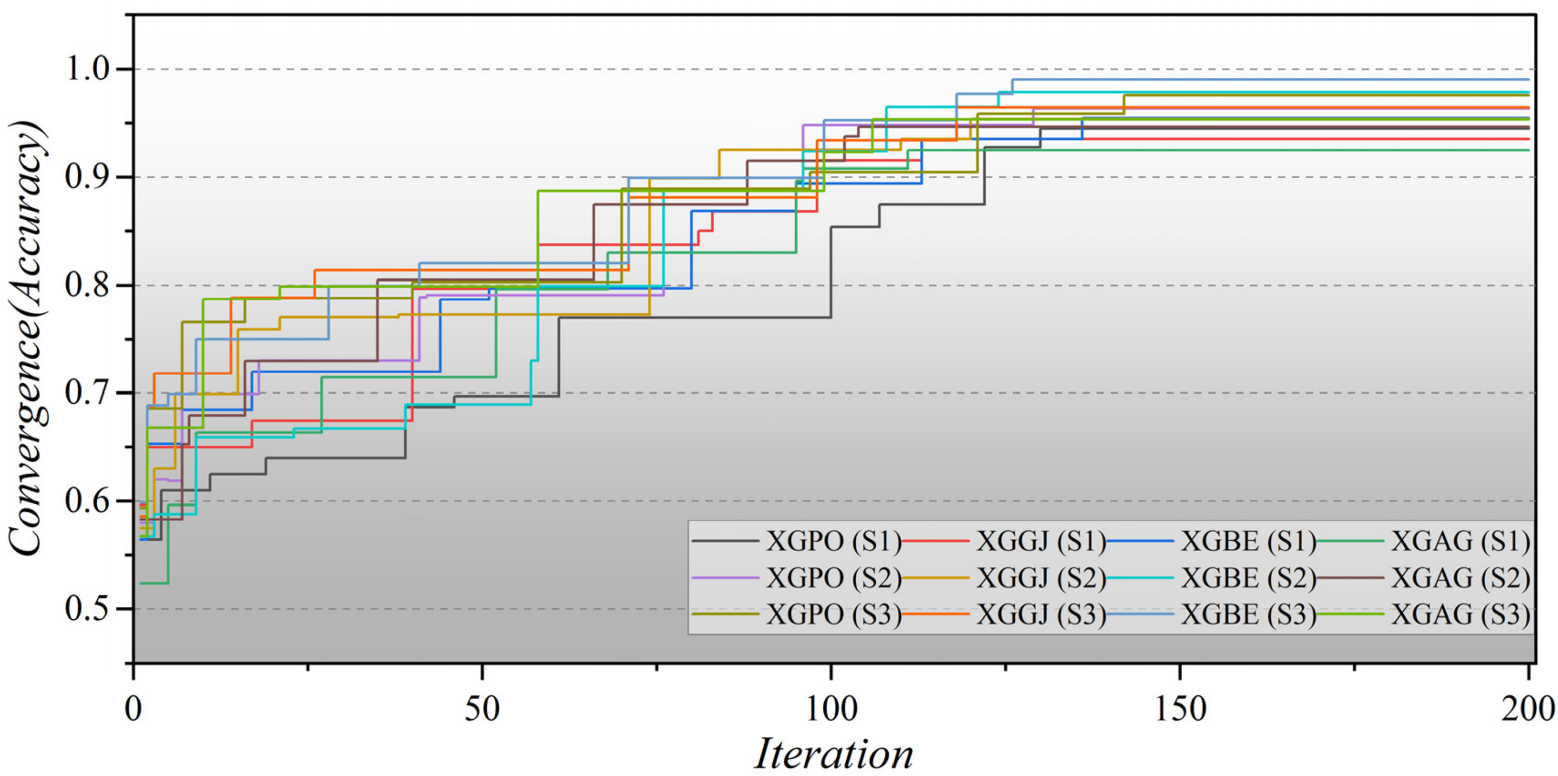
The convergence plot of XGB-based hybrid models in all three scenarios.

Furthermore, Figure 8 illustrates the convergence of hybrid models based on LGB across all three scenarios over 200 iterations. In the second scenario, the initial iterations for the hybrid models commence with a modest Accuracy of approximately 0.5, whereas in the third scenario, they begin with a higher Accuracy of around 0.6. Remarkably, the LGBE (S3) model achieves a remarkable accuracy of 0.97 within approximately 140 iterations. The convergence patterns of XGB-based hybrid models are depicted in Figure 9. Initially, the models display an accuracy of approximately 0.6. The XGBE (S3) model attains an Accuracy of nearly one after 125 iterations. Furthermore, the XGAG (S1) model achieves an Accuracy of 0.91 by the 110th iteration, indicating the weakest performance of features in scenario (1) in training hybrid models.

## 5 Analysis of results

### 5.1 Metrics for evaluating predictions

The importance of performance evaluation criteria in assessing ML algorithms is highlighted in the article, emphasizing the need to select metrics tailored to the specific problem. For comprehensive comparative analysis in classification tasks, widely recognized measures such as Accuracy, Precision, Recall, F1-Score, Correlation Coefficient (MCC), and Heidke Skill Score (HSS) are employed.

Accuracy serves as the primary metric for evaluating the accuracy of predictions. Precision, Recall, and F1-Score complement Accuracy, especially in scenarios with imbalanced data distributions. Precision measures the Accuracy of positive predictions, while Recall identifies all relevant instances within a class. The F1-Score combines both Precision and Recall to provide a balanced assessment. The MCC evaluates the reliability of binary classifications by considering true positives, true negatives, false positives, and false negatives. Higher MCC scores indicate more accurate predictions. MCC is particularly useful for assessing classifiers, especially in cases of unbalanced datasets, as it treats both positive and negative samples equally. These metrics, defined by (Equations 83–87):

(83) $$\mathrm{Accuracy}=\frac{\mathrm{TP}+\mathrm{TN}}{\mathrm{TP}+\mathrm{TN}+\mathrm{FP}+\mathrm{FN}}$$

(84) $$\mathrm{Precision}=\frac{\mathrm{TP}}{\mathrm{TP}+\mathrm{FP}}$$

(85) $$\mathrm{Recall}=\mathrm{TPR}=\frac{\mathrm{TP}}{P}=\frac{\mathrm{TP}}{\mathrm{TP}+\mathrm{FN}}$$

(86) $$F1-\mathrm{Score}\;=\frac{2\times \mathrm{Recall}\;\times \;\mathrm{Precision}}{\mathrm{Recall}+\mathrm{Precision}}$$

(87) $$\mathrm{MCC}=\frac{\mathrm{TP}\times \mathrm{TN}-\mathrm{FP}\times \mathrm{FN}}{\sqrt{\left(\mathrm{TP}+\mathrm{FP})(\mathrm{TP}+\mathrm{FN})(\mathrm{TN}+\mathrm{FP})(\mathrm{TN}+\mathrm{FN}\right)}}$$

TP represents the number of true positives, TN stands for the total of true negatives, FP indicates the number of false positives, and FN denotes the count of false negatives.

The HSS is a statistical metric devised by meteorologist Paul Heidke to evaluate the accuracy of categorical forecasts, primarily in meteorology (83). It involves comparing observed and forecasted categorical outcomes, taking into account hits, correct rejections, false alarms, and misses. The HSS formula provides a comprehensive assessment of predictive skills (Equation 88).

(88) $$\mathrm{HSS}=\frac{2\times (T_{P}F_{N}-F_{P}T_{N})}{\left(T_{P}+T_{N})\times (T_{N}+F_{N})+(T_{P}+F_{P})\times (F_{P}+F_{N}\right)}$$

HSS is a metric used in meteorology to assess the accuracy of categorical weather forecasts. It compares observed and forecasted events. A score of 1 indicates perfect agreement, and 0 suggests performance equivalent to random chance.

### 5.2 Findings and discussion

The results are presented across three scenarios. In the first scenario, the GRACE Scale was applied, incorporating four parameters: HR, Age, SBP, and Killip Class, which are traditionally employed in hospitals (84). Table 5 provides a comprehensive comparison of performance metrics, encompassing Accuracy, Precision, Recall, F1-Score, MCC, and HSS, for the LGBM model alongside its hybrid models (LGAG, LGBE, LGGJ, and LGPO) and the XGBC model with its hybrid versions (XGAG, XGBE, XGGJ, and XGPO) across scenario (1) during both training and testing phases and for all data. Especially, the XGBE model displayed remarkable performance, achieving an Accuracy of 0.954, outperforming other models. Close behind, the LGBE and XGPO models each attained an Accuracy of 0.944. Particular significance was the superior performance demonstrated by the BES optimizer.

**Table 5:** Estimation metrics results for models’ prediction performance based on scenario (1).

Model	Phase	Index values					
		Accuracy	Precision	Recall	F1-score	MCC	HSS
LGBM	Train	0.902	0.877	0.902	0.876	0.446	0.117
	Test	0.890	0.853	0.890	0.868	0.126	
	All	0.893	0.821	0.906	0.862	0.133	
LGAG	Train	0.913	0.908	0.913	0.882	0.298	0.210
	Test	0.924	0.914	0.924	0.902	0.349	
	All	0.916	0.909	0.916	0.888	0.312	
LGBE	Train	0.949	0.946	0.949	0.945	0.677	0.623
	Test	0.932	0.925	0.932	0.927	0.521	
	All	0.944	0.939	0.944	0.940	0.633	
LGGJ	Train	0.926	0.917	0.926	0.916	0.495	0.452
	Test	0.925	0.913	0.925	0.916	0.434	
	All	0.926	0.916	0.926	0.916	0.477	
LGPO	Train	0.940	0.935	0.940	0.936	0.618	0.570
	Test	0.927	0.919	0.927	0.921	0.479	
	All	0.936	0.930	0.936	0.931	0.580	
XGBC	Train	0.914	0.904	0.914	0.908	0.445	0.430
	Test	0.913	0.907	0.913	0.909	0.410	
	All	0.913	0.905	0.913	0.908	0.435	
XGAG	Train	0.923	0.912	0.923	0.908	0.446	0.406
	Test	0.928	0.917	0.928	0.918	0.448	
	All	0.924	0.913	0.924	0.911	0.446	
XGBE	Train	0.959	0.958	0.959	0.957	0.747	0.698
	Test	0.943	0.938	0.943	0.939	0.604	
	All	0.954	0.952	0.954	0.951	0.706	
XGGJ	Train	0.937	0.932	0.937	0.928	0.574	0.521
	Test	0.931	0.921	0.931	0.923	0.486	
	All	0.935	0.928	0.935	0.926	0.548	
XGPO	Train	0.947	0.943	0.947	0.942	0.658	0.620
	Test	0.939	0.933	0.939	0.935	0.571	
	All	0.944	0.940	0.945	0.940	0.633	

In the second scenario, the features selected by SHAP were used, which included ten parameters for the LGBM model and 13 parameters for the XGBC model. Table 6 presents the results of evaluation metrics for the two mentioned single models and their hybrid versions based on scenario (2). The LGBM model was characterized by its relatively lower performance, evidenced by an Accuracy score of 0.921. Conversely, the LGBE model emerged as a standout performer within the domain of LGBM hybrid models, showing notable efficacy with an Accuracy score of 0.963. Especially, the XGBC model displayed the highest level of performance, boasting an impressive Accuracy value of 0.978, thereby establishing itself as the benchmark against which all other models are measured.

**Table 6:** Estimation metrics results for models’ prediction performance based on scenario (2).

Model	Phase	Index values					
		Accuracy	Precision	Recall	F1-score	MCC	HSS
LGBM	Train	0.916	0.922	0.917	0.919	0.550	0.561
	Test	0.933	0.936	0.933	0.934	0.594	
	All	0.921	0.926	0.921	0.923	0.562	
LGAG	Train	0.934	0.936	0.934	0.935	0.632	0.638
	Test	0.942	0.946	0.942	0.943	0.656	
	All	0.936	0.939	0.936	0.937	0.639	
LGBE	Train	0.966	0.968	0.966	0.967	0.817	0.793
	Test	0.957	0.958	0.957	0.957	0.734	
	All	0.963	0.965	0.963	0.964	0.794	
LGGJ	Train	0.947	0.947	0.947	0.947	0.694	0.664
	Test	0.932	0.935	0.932	0.933	0.590	
	All	0.942	0.943	0.942	0.943	0.664	
LGPO	Train	0.961	0.962	0.961	0.961	0.780	0.747
	Test	0.946	0.946	0.946	0.946	0.662	
	All	0.957	0.957	0.957	0.957	0.747	
XGBC	Train	0.944	0.943	0.944	0.943	0.674	0.642
	Test	0.924	0.933	0.924	0.928	0.573	
	All	0.938	0.939	0.938	0.939	0.642	
XGAG	Train	0.950	0.949	0.950	0.949	0.709	0.691
	Test	0.938	0.945	0.938	0.941	0.652	
	All	0.946	0.948	0.946	0.947	0.691	
XGBE	Train	0.981	0.980	0.981	0.980	0.885	0.865
	Test	0.972	0.972	0.973	0.972	0.821	
	All	0.978	0.978	0.978	0.978	0.867	
XGGJ	Train	0.958	0.957	0.958	0.957	0.756	0.732
	Test	0.944	0.950	0.944	0.946	0.682	
	All	0.953	0.955	0.953	0.954	0.733	
XGPO	Train	0.966	0.968	0.966	0.967	0.817	0.793
	Test	0.957	0.958	0.957	0.957	0.734	
	All	0.963	0.965	0.963	0.964	0.794	

The features selected by RFE were applied in the third scenario, comprising six features in the LGBM-based models and eight features in the XGBC-based model. According to Table 7, the XGBE model was the peak performer, boasting an exceptional Accuracy score of 0.990. Following closely, the LGBE model secured the second position with a commendable Accuracy of 0.977, while the XGPO model secured the third rank with an Accuracy score of 0.975. In contrast, the LGBM simple model presented the least impressive performance among the models under analysis.

**Table 7:** Estimation metrics results for models’ prediction performance based on scenario (3).

Model	Phase	Index values					
		Accuracy	Precision	Recall	F1-score	MCC	HSS
LGBM	Train	0.932	0.931	0.932	0.931	0.606	0.610
	Test	0.939	0.940	0.939	0.940	0.621	
	All	0.934	0.937	0.934	0.934	0.610	
LGAG	Train	0.939	0.933	0.939	0.933	0.599	0.594
	Test	0.948	0.964	0.948	0.944	0.636	
	All	0.942	0.936	0.942	0.936	0.609	
LGBE	Train	0.980	0.980	0.980	0.980	0.882	0.864
	Test	0.971	0.971	0.971	0.971	0.818	
	All	0.977	0.977	0.977	0.977	0.864	
LGGJ	Train	0.956	0.953	0.956	0.953	0.723	0.706
	Test	0.956	0.953	0.956	0.953	0.695	
	All	0.956	0.953	0.956	0.953	0.715	
LGPO	Train	0.967	0.966	0.967	0.966	0.801	0.793
	Test	0.965	0.965	0.965	0.965	0.778	
	All	0.966	0.965	0.966	0.966	0.794	
XGBC	Train	0.942	0.940	0.942	0.941	0.653	0.643
	Test	0.938	0.941	0.938	0.939	0.625	
	All	0.941	0.940	0.941	0.940	0.643	
XGAG	Train	0.955	0.955	0.955	0.955	0.741	0.728
	Test	0.949	0.952	0.949	0.950	0.696	
	All	0.953	0.954	0.953	0.953	0.728	
XGBE	Train	0.992	0.991	0.992	0.991	0.951	0.938
	Test	0.986	0.986	0.986	0.985	0.907	
	All	0.990	0.990	0.990	0.990	0.939	
XGGJ	Train	0.965	0.963	0.965	0.963	0.787	0.777
	Test	0.963	0.962	0.963	0.963	0.762	
	All	0.964	0.963	0.964	0.963	0.779	
XGPO	Train	0.980	0.980	0.980	0.980	0.886	0.854
	Test	0.964	0.964	0.964	0.964	0.773	
	All	0.975	0.975	0.975	0.975	0.854	

**Figure 10:**
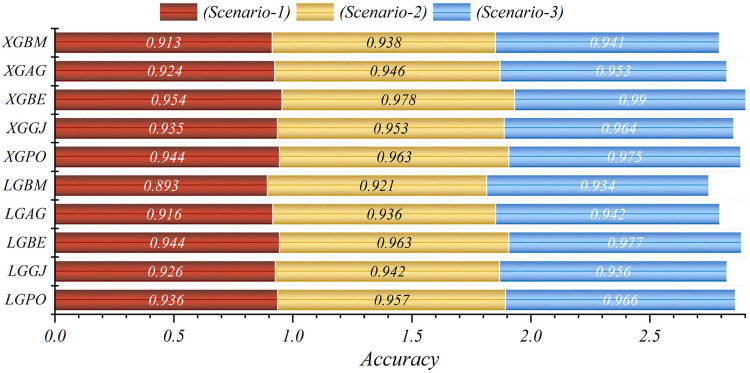
Graphical comparison of accuracy metric for the three scenarios in prediction models.

**Figure 11:**
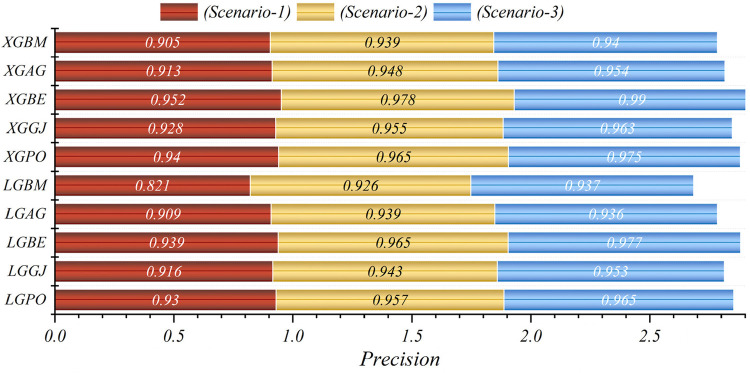
Graphical comparison of precision metric for the three scenarios in prediction models.

**Figure 12:**
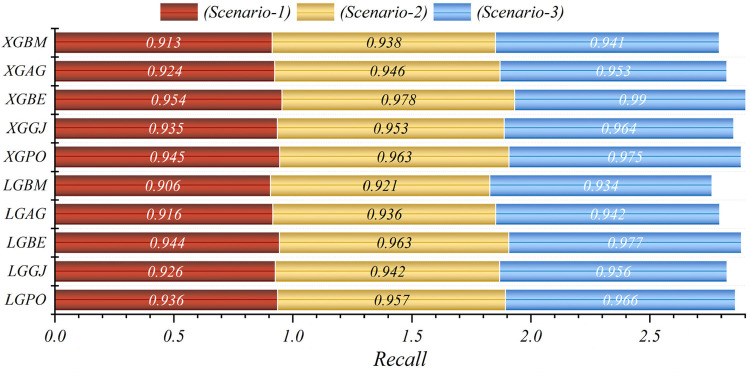
Graphical comparison of recall metric for the three scenarios in prediction models.

**Figure 13:**
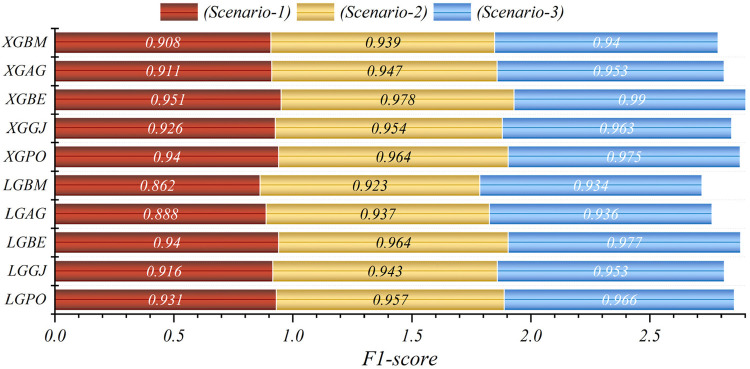
Graphical comparison of F1-score metric for the three scenarios in prediction models.

**Figure 14:**
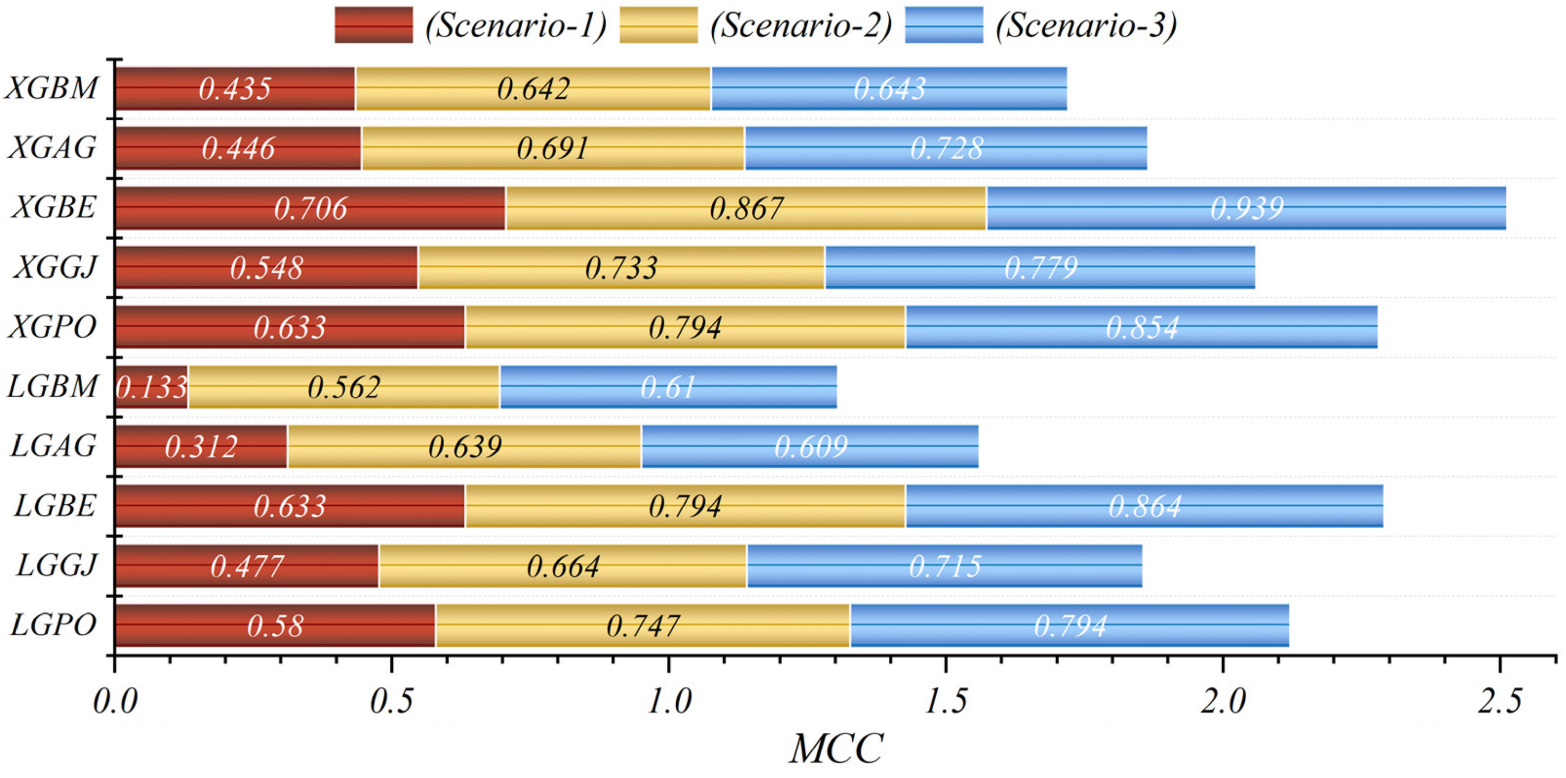
Graphical comparison of MCC metric for the three scenarios in prediction models.

In general, based on the comparative representations presented in Figures 10–14, it is evident that the models from the third scenario outperform those from the first and second scenarios according to the metrics of Accuracy, Precision, Recall, F1-Score, and MCC.

Table 8 displays the evaluation criteria values used to assess the effectiveness of the models in distinguishing between the Alive and Die classes for the first scenario, while Tables 9, 10 present these metric values for the second and third scenarios, respectively.

**Table 8:** The results of the evaluation criteria for assessing the effectiveness of the constructed models in classifying patients in scenario (1).

Model	Phase	Index values		
		Precision	Recall	F1-score
LGBM	Alive	0.301	0.109	0.160
	Die	0.913	0.974	0.943
LGAG	Alive	0.826	0.134	0.230
	Die	0.918	0.997	0.956
LGBE	Alive	0.777	0.563	0.653
	Die	0.956	0.983	0.969
LGGJ	Alive	0.695	0.377	0.489
	Die	0.938	0.983	0.960
LGPO	Alive	0.724	0.518	0.604
	Die	0.952	0.980	0.965
XGBC	Alive	0.551	0.419	0.476
	Die	0.941	0.965	0.953
XGAG	Alive	0.720	0.317	0.440
	Die	0.933	0.987	0.959
XGBE	Alive	0.844	0.630	0.722
	Die	0.963	0.988	0.975
XGGJ	Alive	0.777	0.430	0.553
	Die	0.944	0.987	0.965
XGPO	Alive	0.799	0.546	0.649
	Die	0.955	0.986	0.970

**Table 9:** The results of the evaluation criteria for assessing the effectiveness of the constructed models in classifying patients in scenario (2).

Model	Phase	Index values		
		Precision	Recall	F1-score
LGBM	Alive	0.572	0.641	0.605
	Die	0.962	0.950	0.956
LGAG	Alive	0.648	0.701	0.673
	Die	0.969	0.961	0.965
LGBE	Alive	0.778	0.852	0.813
	Die	0.985	0.975	0.980
LGGJ	Alive	0.687	0.704	0.696
	Die	0.969	0.967	0.968
LGPO	Alive	0.767	0.775	0.771
	Die	0.977	0.977	0.976
XGBC	Alive	0.662	0.690	0.676
	Die	0.968	0.964	0.966
XGAG	Alive	0.702	0.739	0.720
	Die	0.973	0.968	0.970
XGBE	Alive	0.936	0.824	0.876
	Die	0.982	0.994	0.988
XGGJ	Alive	0.739	0.778	0.758
	Die	0.977	0.972	0.974
XGPO	Alive	0.778	0.852	0.813
	Die	0.985	0.975	0.980

**Table 10:** The results of the evaluation criteria for assessing the effectiveness of the constructed models in classifying patients in scenario (3).

Model	Phase	Index values		
		Precision	Recall	F1-score
LGBM	Alive	0.649	0.644	0.647
	Die	0.963	0.964	0.964
LGAG	Alive	0.786	0.518	0.624
	Die	0.952	0.985	0.968
LGBE	Alive	0.891	0.863	0.877
	Die	0.986	0.989	0.987
LGGJ	Alive	0.854	0.637	0.730
	Die	0.963	0.989	0.976
LGPO	Alive	0.856	0.771	0.811
	Die	0.977	0.987	0.982
XGBC	Alive	0.701	0.651	0.675
	Die	0.964	0.971	0.968
XGAG	Alive	0.943	0.764	0.754
	Die	0.976	0.973	0.971
XGBE	Alive	0.970	0.919	0.944
	Die	0.992	0.997	0.994
XGGJ	Alive	0.846	0.754	0.797
	Die	0.975	0.986	0.980
XGPO	Alive	0.869	0.866	0.868
	Die	0.986	0.987	0.986

In all three scenarios, the models demonstrated higher accuracy in predicting and classifying patients in the Die class compared to the Alive class. Comparing the performance of the models in the Alive class in the first scenario, the XGBE model displayed superior performance with a Precision of 0.844, representing a 12.36% decrease compared to its Precision in the Die class. Conversely, the LGBE model outperformed the LGPO model with a Precision of 0.777. Moving to the second and third scenarios, the XGBE model achieved Precision values of 0.936 and 0.970, respectively, showing improved performance by 9.83% and 12.99% compared to the first scenario. Furthermore, the LGBE model maintains consistent performance in the second scenario, with a marginal difference of 0.13%, while in the third scenario, it demonstrated superior performance with a 12.79% increase.

In the first scenario, the XGBE model achieved the maximum performance in the Die class with a Precision of 0.963, while the LGBE, XGPO, and LGPO models displayed nearly identical performance in this class, with Precision values of 0.956, 0.955, and 0.952, respectively. Moving to the second scenario, the XGPO model demonstrated superior performance in classifying patients in the Die class with a Precision of 0.985, while the XGBE model ranked third with a slight difference of 0.31%. Lastly, in the third scenario, the XGBE model surpassed all others with an impressive Precision of 0.992 in the Die class, securing the top position. The LGBE model followed closely behind with a Precision of 0.986, earning the second rank.

**Figure 15:**
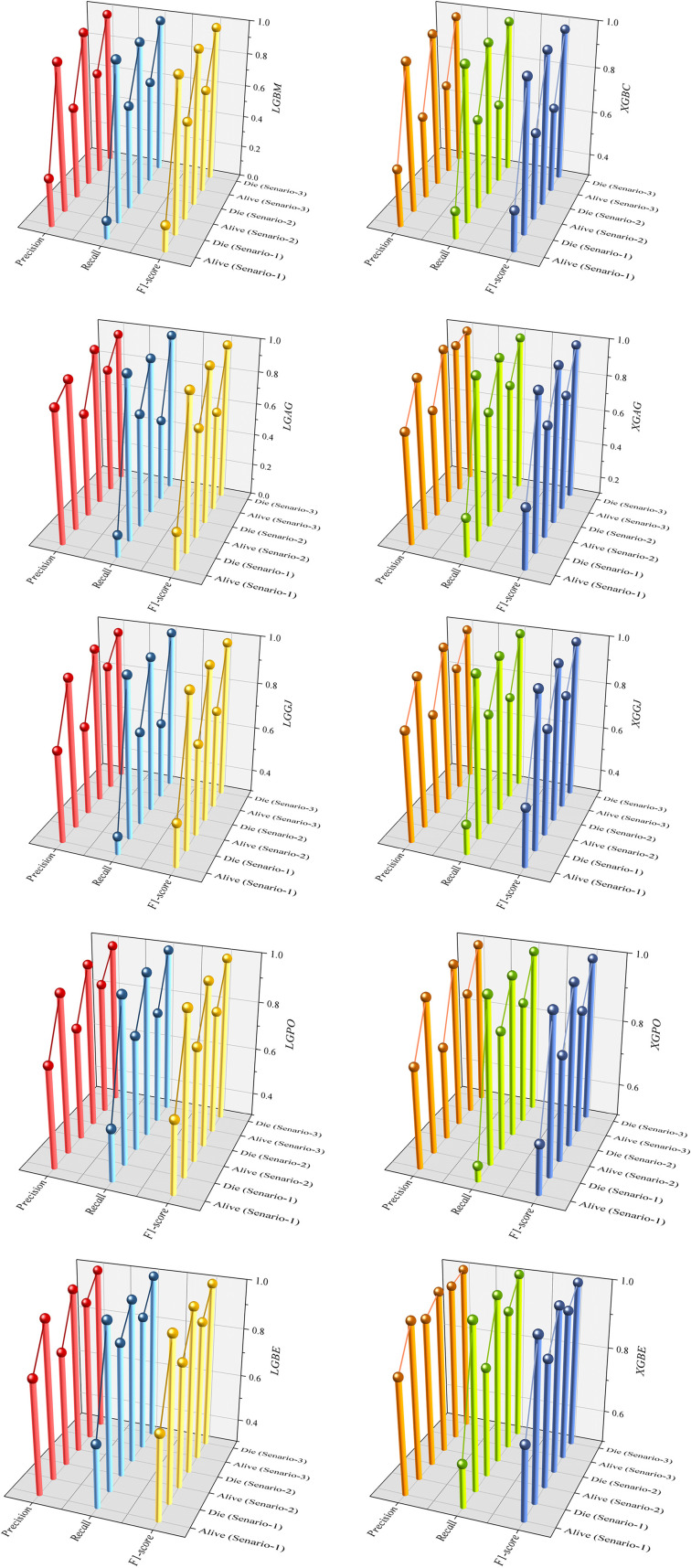
Comparative visual display of evaluation metrics for models across three scenarios in the Die and alive classes.

Figure 15 presents a visual comparison of the models introduced in this research across scenarios (1), (2), and (3), using Precision, Recall, and F1-score metrics. In the LGBM and XGBC basic models, the Recall values are lower than those of other hybrid models in the Alive class, with values of 0.109 and 0.419 for the first scenario, 0.641 and 0.690 for the second scenario, and 0.644 and 0.651 for the third scenario, respectively. The lowest Recall value is attributed to the LGBM model in scenario (1) for the classification of Alive patients, while the highest value is recorded for the XGBE model in the third scenario and LGAG in the first scenario in the Die class, both with a value of 0.997.

**Figure 16:**
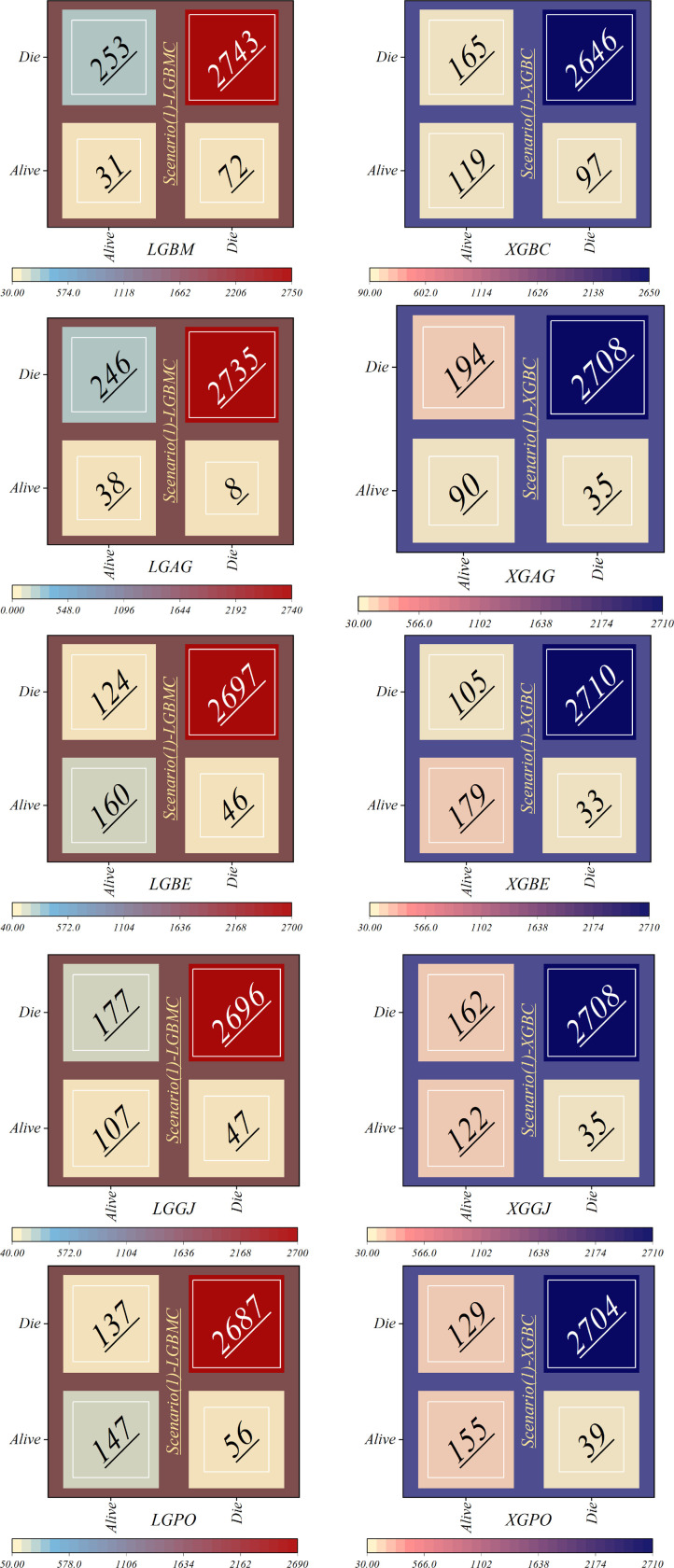
Confusion matrices depicting the accuracy of individual models within scenario (1).

Figure 16 displays the confusion matrix depicting the classification performance in scenario (1), using the four features introduced by the GRACE Scale. This visual representation offers insights into the model's classification outcomes across various diagnostic categories. The LGBM model showed the highest error rate in misclassifying individuals from the Alive class into the Die group, with 253 patients misclassified. Following closely, the LGAG model ranked next, committing a similar error with 246 misclassified patients. Conversely, the LGAG model demonstrated the lowest error rate, misclassifying only eight deceased patients into the Alive class.

Additionally, the XGBC model incorrectly classified 97 dead patients into the Alive group. In contrast, the LGBE model showcased superior performance compared to other hybrid models based on LGBM, with 124 and 46 misclassifications in the Alive and Die classes, respectively. Similarly, the XGBE model exhibited the lowest misclassification rate compared to other XGBC-based hybrid models.

**Figure 17:**
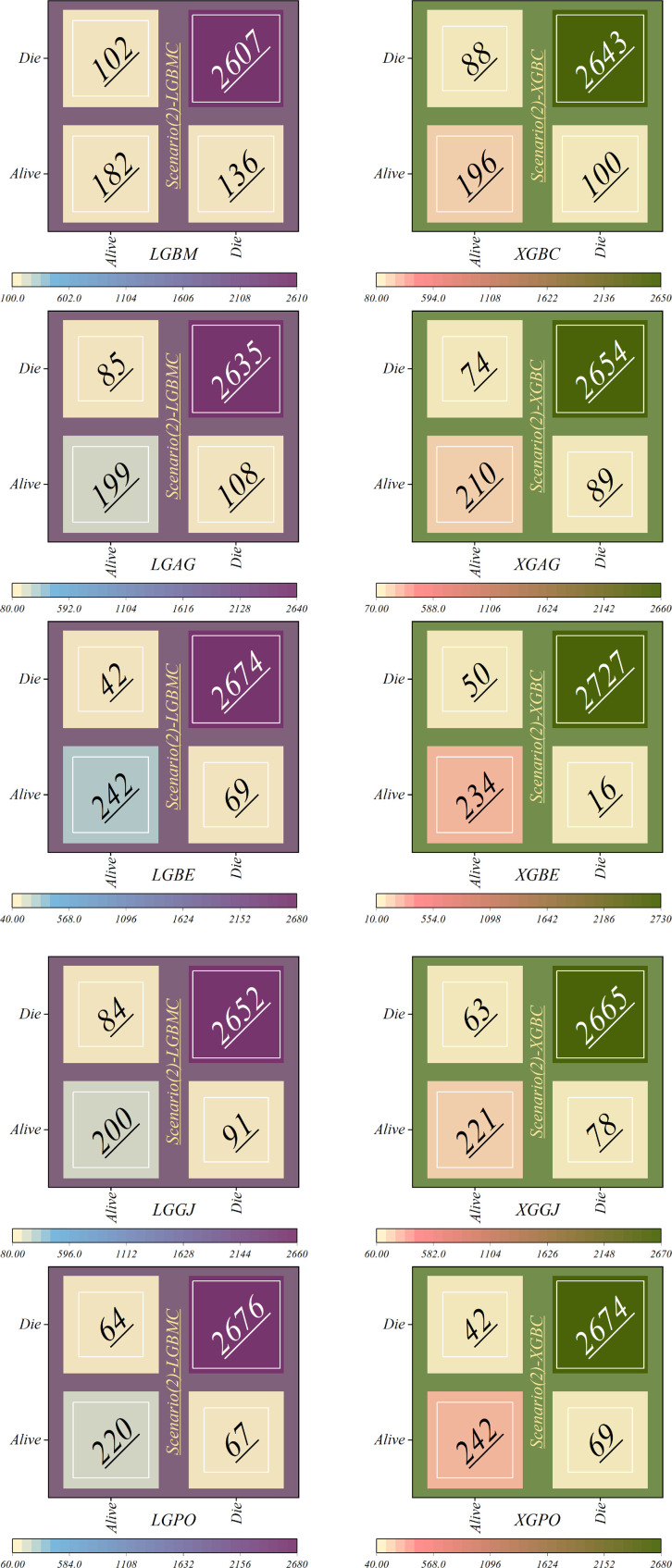
Confusion matrices depicting the accuracy of individual models within scenario (2).

**Figure 18:**
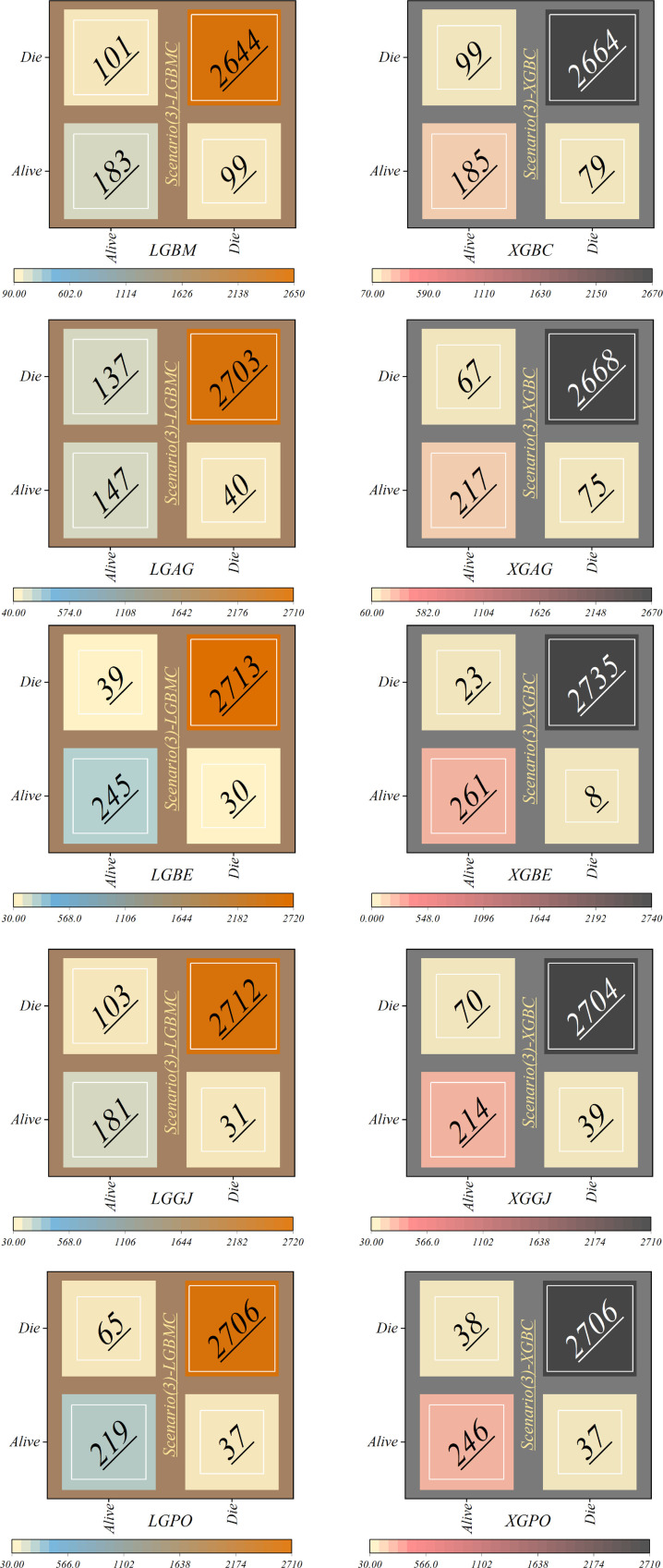
Confusion matrices depicting the accuracy of individual models within scenario (3).

Figure 17 depicts the correct and incorrect classification results of the models based on scenario (2), while Figure 18 represents those based on scenario (3). In the second scenario, SHAP was employed to identify effective features in modeling, whereas the third scenario employed RFE, resulting in an obvious increase in model accuracy. In scenario (2), as illustrated in Figure 17, the LGBM model continued to display the highest misclassification rate in the Alive class, speciously placing 102 patients in the Die class; however, it had enhanced its performance by 59.68% in correctly classifying the group of living patients. Conversely, the LGBE and XGPO models demonstrated the lowest errors in classifying living patients, misclassifying only 42 patients while correctly classifying 242 patients. The XGBE model excelled in classifying dead individuals, accurately classifying 2,727 patients while misclassifying only 16 patients.

In scenario (3), as delineated in Figure 18, notable discrepancies appear in the classification of alive patients. Specifically, the LGAG model shows a significant degree of error, misclassifying 137 patients. Similarly, the LGBM model demonstrates a considerable level of misclassification, with 99 patients incorrectly assigned to the Alive class. Contrarily, the XGBE model displays admirable performance, achieving 261 correct classifications and 23 misclassifications within the Alive group. Impressively, the XGBE model makes minimum errors, with only eight deceased patients erroneously categorized as Alive.

In general, the models in scenario (1) show the weakest performance, while the highest performance is observed in the third scenario. The application of scenario (1) in hospitals entails a high risk, as it relies only on four features: HR, Age, SBP, and Killip Class. Conversely, in scenario (2), the models employ ten features for LGBM and 13 features for XGB, leading to significantly higher accuracy compared to predictions based on the GRACE score. In scenario (3), the efficiency of the models surpasses that of scenarios (1) and 2 despite using fewer features 6 for the LGBM model and 8 for the XGB model. It is noteworthy that despite the reduced number of parameters, higher accuracy has been achieved. Upon comparing the two models, it can be concluded that the XGBE model offered the highest accuracy with eight features. This level of accuracy allows hospitals and healthcare professionals to predict the probability of survival more accurately, thereby reducing in-hospital mortality rates and tailoring treatments accordingly.

On the other hand, scenario (3) demanded a diminished set of parameters in comparison to scenario (2), thereby reducing the time required for testing. Such efficiency is particularly admirable in the context of patients’ serious conditions, where timely intervention is paramount. Moreover, the efficient testing regimen of scenario three not only hastens decision-making but also mitigates financial burdens. The decreased number of requisite tests translates to lower costs incurred by both patients and healthcare facilities, emphasizing the compelling value proposition of the model's heightened accuracy.

**Figure 19:**
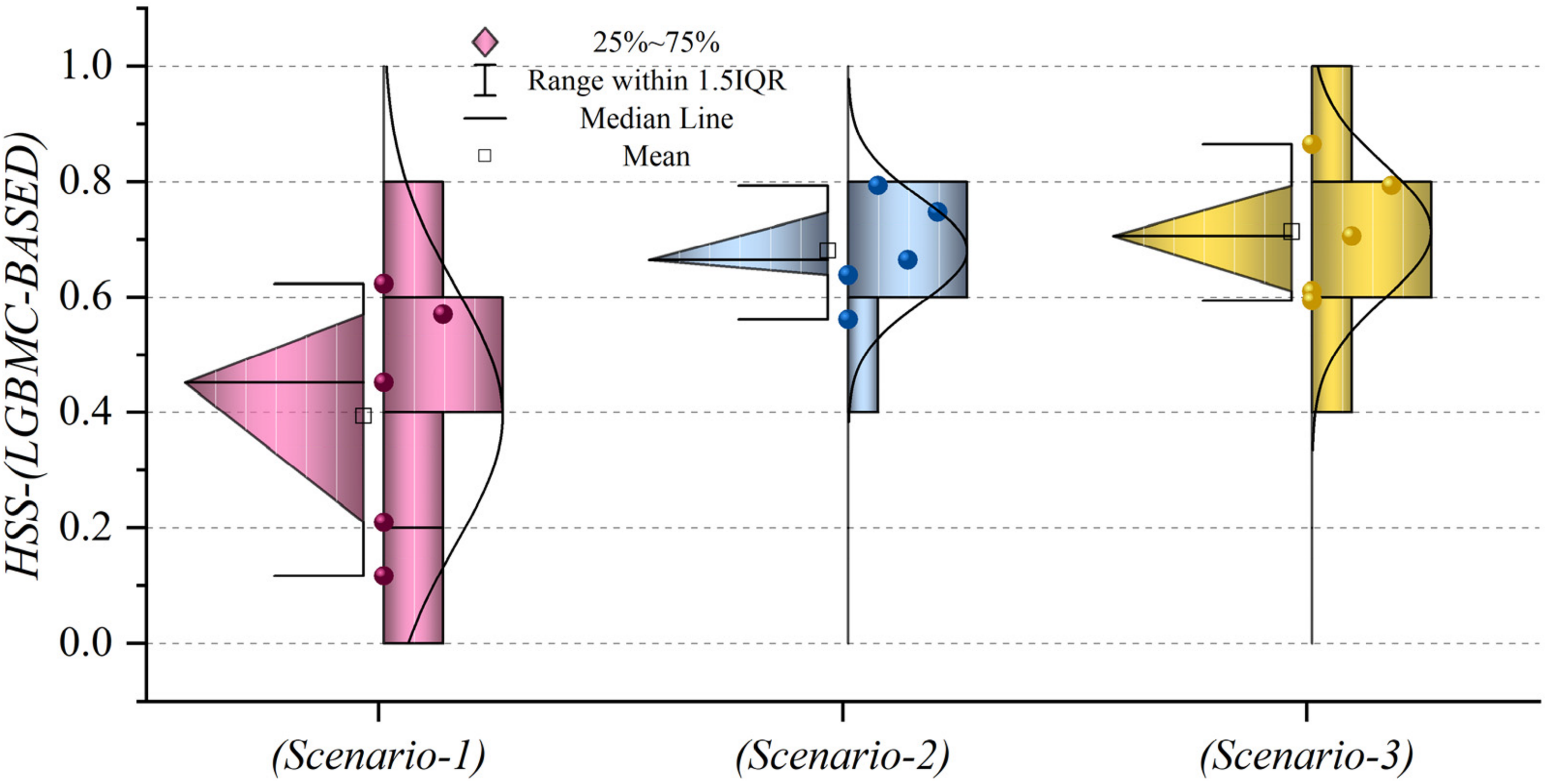
The chart illustrates the HSS values of LGBM models across three scenarios.

**Figure 20:**
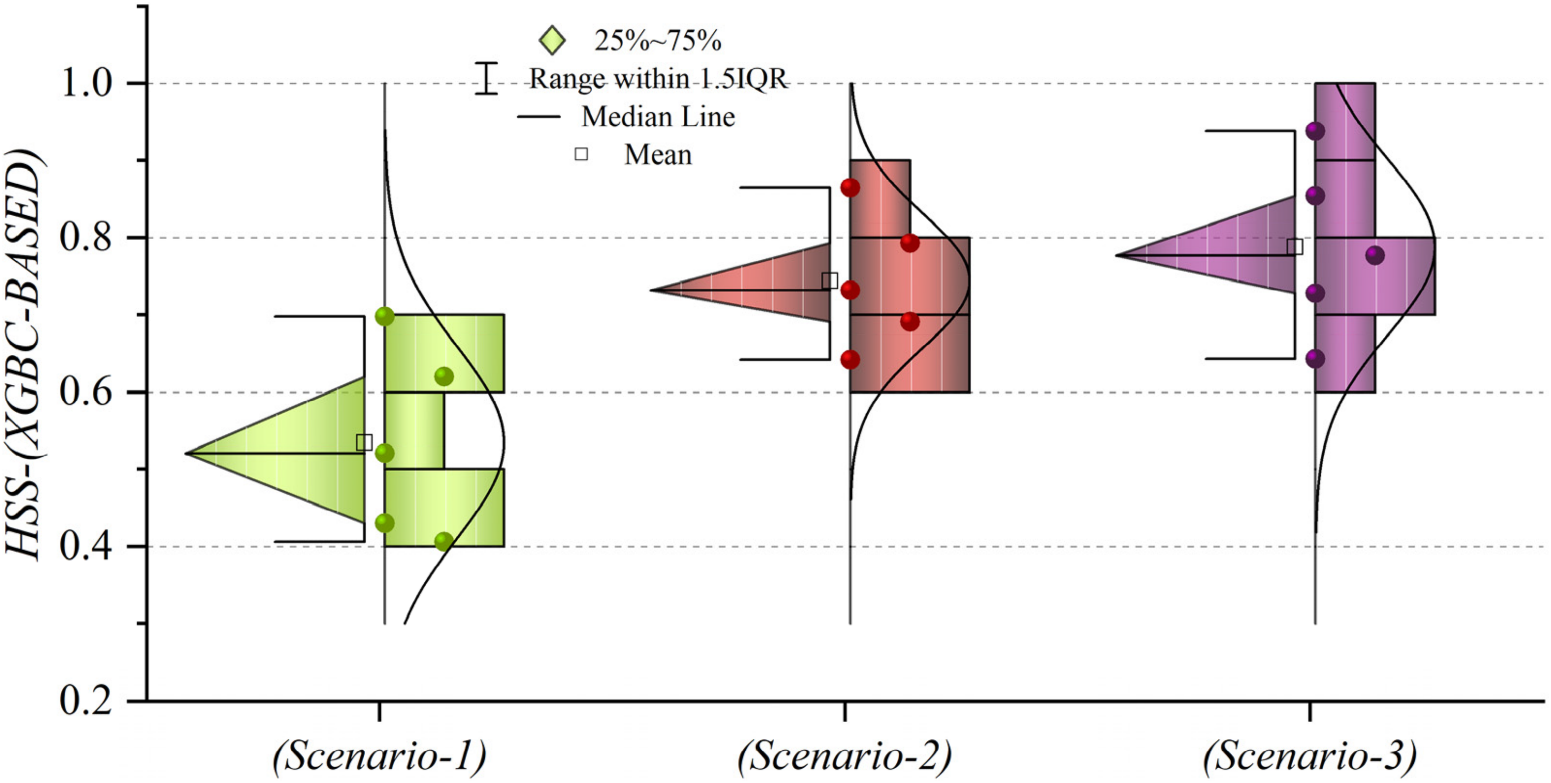
The chart illustrates the HSS values of XGBC models across three scenarios.

Figures 19, 20 depict HSS values for models based on LGBM and XGBC, respectively, to assess the accuracy of the predictions. In Figure 19, the mean HSS value for the third scenario approximates 0.7, while for the second scenario, it is around 0.65. Notably, the overall mean HSS value for the first scenario is approximately 0.4. This delineates that in scenario (1), the predictive accuracy stands at roughly 40%, which deviates from acceptable performance standards. Conversely, as depicted in Figure 20, the mean HSS value is about 0.5, highlighting the models’ lack of precision in scenario one concerning patient prediction and classification accuracy. Moreover, the mean HSS value for XGBC-based models in scenarios (2) and (3) averages approximately 0.67 and 0.71, respectively. Collectively, these findings prove the superior performance of models in scenario (3), revealing their exceptional forecasting capabilities and optimal operational efficiency.

### 5.3 Comparative analysis

In this section, for comparing the Accuracy of predictions conducted by the best developed model (XGBE in the third scenario) in the study by those models in existing literature, the metric results are reported in Table 11. The results reveal that the Accuracy, Precision, and F1-score of the XGBE were 3% to 5% higher than the developed Catboost in the previous study.

**Table 11:** Comparison results between the accuracy of the best developed model with models in existing literature.

Developed model	Reference	Accuracy	Precision	F1-score
Categorical boosting (Catboost)	(32)	0.96	0.95	0.97
XGBE (XGB optimized with BEO)	This study	0.99	0.99	0.99

## 6 Conclusion

Cardiovascular disease presents a significant global health challenge, especially in low-income countries, contributing to increased mortality rates. Myocardial infarction (MI) arises from reduced blood flow to the heart, leading to tissue damage and symptoms like chest pain and shortness of breath. Effective management of ST-segment elevation myocardial infarction (STEMI) was critical, with early reperfusion therapy, particularly through percutaneous coronary intervention (PCI), prioritized for optimal care. This study employed advanced machine learning (ML) techniques to investigate risk factors influencing in-hospital mortality (IHM) in MI patients following PCI. Many ML classifiers, such as Extreme Gradient Boosting (XGB), Light Gradient Boosting (LGB), Stochastic Gradient Boosting (SGB), and Histogram Gradient Boosting (HGB), were used, and Monte Carlo cross-validation (MCCV) assisted in selecting top-performing models. Three scenarios were designed to evaluate forecast accuracy, one of which (scenario 1) was based on the traditional GRACE scaling system which can be calculated using online calculators available on medical websites or through electronic health record systems. The objective of this study was to provide insights to improve risk assessment and patient care strategies for MI patients undergoing PCI by using more imperative features of the patients rather than those utilized in traditional methods (GRACE score), which are extracted by feature selection methods. Additionally, meta-heuristic algorithms, including Gray Wolf Optimizer (AGWO), Bald Eagle Search Optimization (BES), Golden Jackal Optimizer (GJO), and Puma Optimizer (PO), were employed to enhance prediction accuracy.

In the evaluation of scenario (1) using the F1-Score standard, the LGBE and XGBE models demonstrated superior performance with values of 0.940 and 0.951, respectively. In the second scenario, these values increased to 0.964 and 0.978, indicating an improvement of 2.4% and 2.76% in model performance. Moreover, in scenario (3), these models showed further performance enhancements, with F1-score values increasing by 3.79% and 3.9%. The MCC value for the LGBE and XGBE models in the third scenario reached the highest level, with scores of 0.864 and 0.939, respectively. Despite scenario (1)'s reliance on only four features and its consequent weak performance, scenarios (2) and (3) demonstrate improved accuracy by applying more parameters. Especially, scenario (3) surpasses the others in efficiency despite employing fewer features, with the XGB model achieving the highest accuracy using eight features. This improved accuracy enables hospitals to predict survival probabilities more precisely, thereby reducing in-hospital mortality rates and permitting tailored treatments. Scenario (3)'s streamlined parameter testing process makes it the preferred choice, offering swift decision-making and cost reductions while ensuring accurate forecasts, particularly critical in serious patient conditions. Furthermore, the model constructed in this study can be integrated into clinical decision support systems, such as electronic health record (EHR) systems, to automatically provide risk scores when assessing STEMI patients, assisting doctors in considering the patient's IHM risk when choosing treatment strategies. Thus, a personalized treatment plan can be developed based on the patient's IHM risk level. For example, in high-risk patients, more proactive preventive treatment measures, such as early cardiac rehabilitation programs or intensified medication therapy, can be considered. At the same time, the predictive results of the model can serve as a basis for discussion among multidisciplinary teams, promoting communication and collaboration among medical personnel with different professional backgrounds, and jointly developing the best treatment plan for the patient.

## 7 Limitations

The main limitation of this study is the single-center nature of the data source, which may limit the assessment of the model's generalizability. Additionally, although we have established an effective predictive model, we have not conducted detailed analyses on different patient subgroups, which may affect the model's applicability within specific subgroups. Future studies will address these limitations by collecting multicenter data and performing subgroup analyses to improve the model's generalizability and accuracy.

## Data Availability

The original contributions presented in the study are included in the article/Supplementary Material, further inquiries can be directed to the corresponding author.
