# Supplementary material for: Prediction of in-hospital mortality risk for patients with acute ST-elevation myocardial infarction after primary PCI based on predictors selected by GRACE score and two feature selection methods
Source: Front Cardiovasc Med. 2024 Oct 22;11:1419551. doi: 10.3389/fcvm.2024.1419551 (PMC11534735; doi:10.3389/fcvm.2024.1419551)
Supplement: Supplementary file 1 [file Datasheet1.pdf]

Supplementary Material:

The related codes for the XGBE hybrid model:

- **BESO:**

```
import numpy as np
import time

def BES(nPop, MaxIt, low, high, dim, fobj):
    start_time = time.process_time()

    BestSol = structure(cost=-np.inf, pre=None, position=None)

    # Initialize population
    pop = [Solution() for _ in range(nPop)]
    for i in range(nPop):
        pop[i].pos = low + (high - low) * np.random.rand(dim)
        pop[i].cost = fobj(pop[i].pos)
        if pop[i].cost < BestSol.cost:
            BestSol.pos = pop[i].pos.copy()
            BestSol.cost = pop[i].cost

    print(f"0 {BestSol.cost}")
    Convergence_curve = np.zeros(MaxIt)

    for t in range(MaxIt):
        # 1. Select space
        pop, BestSol, s1 = select_space(fobj, pop, nPop, BestSol, low, high, dim)
        # 2. Search in space
        pop, BestSol, s2 = search_space(fobj, pop, BestSol, nPop, low, high)
        # 3. Swoop
        pop, BestSol, s3 = swoop(fobj, pop, BestSol, nPop, low, high)

        Convergence_curve[t] = BestSol.cost
        print(f"{t + 1} {BestSol.cost}")

    end_time = time.process_time()
    timep = end_time - start_time

    return BestSol, Convergence_curve, timep

def select_space(fobj, pop, nPop, BestSol, low, high, dim):
    Mean = np.mean([ind.pos for ind in pop], axis=0)
```

```
    lm = 2
    s1 = 0

    for i in range(nPop):
        newsol = Solution()
        newsol.pos = BestSol.pos + lm * np.random.rand(dim) * (Mean - pop[i].pos)
        newsol.pos = np.clip(newsol.pos, low, high)
        newsol.cost = fobj(newsol.pos)

        if newsol.cost < pop[i].cost:
            pop[i].pos = newsol.pos
            pop[i].cost = newsol.cost
            s1 += 1
            if pop[i].cost < BestSol.cost:
                BestSol.pos = pop[i].pos.copy()
                BestSol.cost = pop[i].cost

    return pop, BestSol, s1

def search_space(fobj, pop, best, nPop, low, high):
    Mean = np.mean([ind.pos for ind in pop], axis=0)
    a = 10
    R = 1.5
    s1 = 0

    pop = np.random.permutation(pop)
    x, y = polr(a, R, nPop)

    for i in range(nPop - 1):
        newsol = Solution()
        Step = pop[i].pos - pop[i + 1].pos
        Step1 = pop[i].pos - Mean
        newsol.pos = pop[i].pos + y[i] * Step + x[i] * Step1
        newsol.pos = np.clip(newsol.pos, low, high)
        newsol.cost = fobj(newsol.pos)

        if newsol.cost < pop[i].cost:
            pop[i].pos = newsol.pos
            pop[i].cost = newsol.cost
            s1 += 1
            if pop[i].cost < best.cost:
                best.pos = pop[i].pos.copy()
                best.cost = pop[i].cost

    return pop, best, s1
```

```
def swoop(fobj, pop, best, nPop, low, high):
    Mean = np.mean([ind.pos for ind in pop], axis=0)
    a = 10
    R = 1.5
    s1 = 0

    pop = np.random.permutation(pop)
    x, y = swoo_p(a, R, nPop)

    for i in range(nPop):
        newsol = Solution()
        Step = pop[i].pos - 2 * Mean
        Step1 = pop[i].pos - 2 * best.pos
        newsol.pos = np.random.rand(len(Mean)) * best.pos + x[i] * Step + y[i] * Step1
        newsol.pos = np.clip(newsol.pos, low, high)
        newsol.cost = fobj(newsol.pos)

        if newsol.cost < pop[i].cost:
            pop[i].pos = newsol.pos
            pop[i].cost = newsol.cost
            s1 += 1
            if pop[i].cost < best.cost:
                best.pos = pop[i].pos.copy()
                best.cost = pop[i].cost

    return pop, best, s1

def swoo_p(a, R, N):
    th = a * np.pi * np.exp(np.random.rand(N))
    r = th
    xR = r * np.sinh(th)
    yR = r * np.cosh(th)
    xR = xR / np.max(np.abs(xR))
    yR = yR / np.max(np.abs(yR))
    return xR, yR

def polr(a, R, N):
    th = a * np.pi * np.random.rand(N)
    r = th + R * np.random.rand(N)
    xR = r * np.sin(th)
    yR = r * np.cos(th)
    xR = xR / np.max(np.abs(xR))
    yR = yR / np.max(np.abs(yR))
    return xR, yR

class Solution:
```

```
    def __init__(self):
        self.pos = None
        self.cost = float('inf')

# Example usage:
# Define a sample objective function


# Set parameters
nPop = 50
MaxIt = 200
low = 1
high = 1000
dim = 7
```

- **XGBC:**

```
import numpy as np
from xgboost import XGBClassifier
from sklearn.metrics import accuracy_score
from ypstruct import structure

def objective_function(pop):
        net = XGBClassifier(
                n_estimators=333,
                max_depth=371,
                learning_rate=0.324,
                colsample_bytree=0.578,
                subsample=0.484,
                reg_alpha=0.143,
                reg_lambda=0.143
                )
        net.fit(X_tr, y_tr)
        preds_train = net.predict(X_tr)
        preds_val = net.predict(X_te)

        if preds_train.ndim == 2 and preds_train.shape[1] == 1:
            preds_train = preds_train.flatten()


        if preds_val.ndim == 2 and preds_val.shape[1] == 1:
            preds_val = preds_val.flatten()

        y = np.hstack([y_tr, y_te])
        preds = np.hstack([preds_train, preds_val])
```

```
acc_in = accuracy_score(y, preds)
acc_in_tr = accuracy_score(y_tr, preds_train)
acc_in_te = accuracy_score(y_te, preds_val)
POP = structure(cost=acc_in, pre=preds, position=pop)
```
